# Disease-Associated Mutations Impact DNA Methyltransferase 1 Function through Dynamic Allosteric Effects and Solvent Exposure

**DOI:** 10.1021/acs.jctc.6c00982

**Published:** 2026-08-21

**Authors:** Song Xie, Can Bora Yildiz, Paolo Ruggerone, Ke Zuo, Geraldine Zimmer-Bensch, Paolo Carloni

**Affiliations:** † 196554 Institute of Neuroscience and Medicine (INM-9) Computational Biomedicine, Forschungszentrum Jülich GmbH, Jülich 52428, Germany; ‡ RWTH Aachen University, Department of Physics, Aachen 52074, Germany; § 9165 RWTH Aachen University, Department of Neuroepigenetics, Institute of Zoology (Biology 2), Worringerweg 3, 52074 Aachen, Germany; ∥ Department of Physics, 3111 University of Cagliari, Cittadella Universitaria, S.P.monserrato-Sestu Km 0.700, Monserrato (CA) I-09044, Italy; ⊥ College of Pharmacy (International Academy of Targeted Therapeutics and Innovation), Chongqing University of Arts and Sciences, Chongqing 402160, PR China

## Abstract

Mutations in the DNA methyltransferase 1 (DNMT1) enzyme can lead to neurodegenerative diseases, including autosomal dominant cerebellar ataxia–deafness and neuropathy (ADCA-DN) and hereditary sensory and autonomic neuropathy type 1E (HSAN1E). The impact of these mutations on the structural dynamics of DNMT1 remains largely unknown. Here, we present an extensive molecular dynamics investigation of wild-type (WT) DNMT1 and its mutants associated with ADCA-DN (A554V, G589A, and V590F) and HSAN1E (D490E-P491Y and Y495C). The first group of mutants increases structural flexibility and disrupts the allosteric communication relative to WT DNMT1. These findings provide a molecular explanation for the reduced *in vitro* thermostability observed in these mutants. The second group has a similar impact on structural flexibility and allosteric communication and increases solvent exposure at mutant sites, offering a plausible structural explanation for the experimentally observed aberrant protein degradation and cleavage. Furthermore, all the investigated variants alter the most significant large-scale motions of the enzyme. This disruption of motion, together with decreased structural stability, may affect DNMT1’s molecular recognition processes with its cellular partners. Overall, this study sheds light on the molecular mechanisms underlying ADCA-DN and HSAN1E, which could inform the development of therapies.

## Graphical abstract

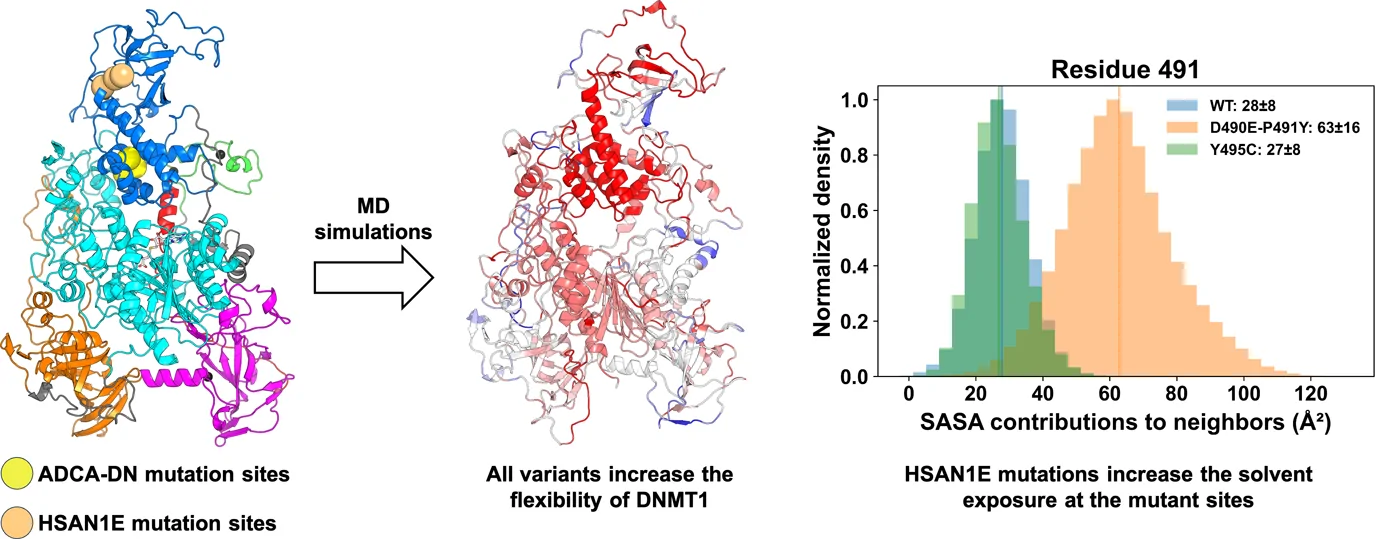

## 1 Introduction

Human DNA methyltransferases (DNMTs) transfer a methyl group from the S-adenosyl-L-methionine (SAM) cofactor to the C5 position of cytosine, thereby producing 5-methylcytosine. DNMT1, encoded by the *DNMT1* gene, is essential for maintaining DNA methylation patterns by preferentially methylating hemimethylated CpG sites.^1^ The enzyme also contributes to *de novo* methylation, which underlies long-term gene regulation and the proper establishment of neuronal circuits.^2^ Our research and that of others have demonstrated that DNMT1 plays roles beyond its canonical function of maintaining DNA methylation, including processes in non-dividing cells, such as neurons. It regulates cytoskeletal organization, neuronal migration, survival, and neuronal morphogenesis through various mechanisms, including interactions with histone modifications and cytosolic actions, in addition to DNA methylation.^2–7^

The enzyme is a multidomain protein consisting of an N-terminal disordered region (NDR), a replication foci-targeting sequence (RFTS) domain, a CXXC zinc finger domain, an autoinhibitory linker (AILK), two bromo-adjacent homology domains (BAH1 and BAH2), and a C-terminal catalytic domain (CAT) (Figure S1).^8^

Several mutations of the protein are associated with cancers and neurodegenerative diseases.^9,10^ As many as thirty-eight of them (Figure S1) are associated with the latter, including autosomal dominant cerebellar ataxia–deafness and neuropathy (ADCA-DN) and hereditary sensory and autonomic neuropathy type 1E (HSAN1E). They have long represented a conceptual challenge in neurodegeneration research because they affect a ubiquitously required DNA methyltransferase, best known for its role in maintaining methylation during cell division.^1^ Yet, pathogenic DNMT1 variants manifest as late-onset, relatively selective degeneration of postmitotic neurons rather than a global loss-of-function phenotype.^11^

Almost all of these mutations (34 out of 38) cluster within the RFTS domain [The remaining few mutations are distributed across the NDR (n = 1), BAH1 (n = 1), and CAT (n = 2) domains (Figure S1)], which plays a pivotal regulatory role by governing DNA access to the CAT domain (Figure S1).^8^ Sub-microsecond all-atom molecular dynamics (MD) simulations have uncovered allosteric communication between the CAT and RFTS domains.^12^ The latter regulates the recruitment of DNMT1 to replication-associated methylation sites through interactions with ubiquitin-like proteins containing PHD and RING finger domains 1 (UHRF1) and/or ubiquitinated histone H3 (H3ub2).^13^ Therefore, perturbations in the structural dynamics of the RFTS domain may affect DNMT1 function.

Several experiments have been performed on disease-linked DNMT1 variants (see Table S1 for details). Among these, *in vitro* biochemical studies have shown that A554V, G589A, and V590F DNMT1 (associated with ADCA-DN) lead to a decreased melting temperature (*T*_m_) and specific destabilization of the RFTS domain.^14,15^ In addition, cell-based assays have shown that D490E-P491Y and Y495C DNMT1 (associated with HSAN1E) feature increased protein degradation^16^ and cleavage by proteases (absent in wild-type (WT) DNMT1).^17^

Here, in an effort to provide a structural basis for these experiments, we investigate WT DNMT1 and the aforementioned protein variants using extensive MD simulations. First, we perform 4 independent 2.5-microsecond all-atom simulations of WT DNMT1 in complex with its endogenous ligand, SAM, in solution, and of the variants that destabilize DNMT1 (A554V, G589A, and V590F; Table S1 and S2; see section Materials and Methods for details). Using distance-fluctuation (DF)^18^ and allosteric communication^19^ analyses of the collected MD trajectory, we demonstrate that three mutations destabilize the enzyme by increasing structural flexibility and hampering allosteric communication.

Next, we use the same protocol as above to offer structural insights into the two mutations (D490E-P491Y and Y495C; Table S1 and S2; see section Materials and Methods for details) that cause aberrant protein cleavage and degradation in cells.^16,17^ Of course, comparisons between *in silico* models and cellular outcomes should be interpreted with a grain of salt, as *in silico* models mimic *in vitro* conditions more properly (as opposed to in cells). By performing the analysis above [along with solvent-accessible surface area (SASA)] on the MD trajectories, we predict that both variants similarly increase structural flexibility and weaken allosteric communication, thereby potentially enabling the protein to adopt conformations that are more favorable for protein degradation. Additionally, increased local exposure to solvents at the mutant sites observed in both variants may contribute to abnormal protein cleavage.

We further conducted principal component analysis (PCA) to investigate the impact of mutations on the large-scale motions of DNMT1. The results indicate that all variants significantly disrupt the opening movement of the N-lobe in the RFTS domain observed in WT DNMT1 (PC1). By comparing disrupted large-scale motions and the observed structural flexibility with existing experimental and computational data, we further propose implications for the potential impact of these variants on DNMT1 proteomics and associated functions.

Overall, this work provides a molecular basis for the *in vitro* structural effects of three ADCA-DN mutations and for the cellular defects observed in two HSAN1E mutations. They might help elucidate the mechanisms underlying these two neurodegenerative diseases, thereby guiding future therapeutic strategies.

## 2 Materials and Methods

### 2.1 MD Simulations

MD simulations are performed on WT DNMT1/variants in complex with SAM in solution. Specifically, the initial protein structures are constructed using the Swiss-Model web server,^20^ with the crystal structure (PDB ID 4WXX^8^) as the template. The structural information for residues 1–350 is not represented in any of the DNMT1 structures that have been experimentally determined using X-ray crystallography or cryo-electron microscopy (PDB IDs: 3AV4,^20^ 3AV5,^20^ 3AV6,^20^ 3PT6,^21^ 3PT9,^21^ 3PTA,^21^ 4DA4,^22^ 4YOC,^23^ 4WXX,^8^ 5GUV,^24^ 5GUT,^24^ 5WY1,^25^ 6W8V,^26^ 6W8W,^26^ 6X9I,^27^ 6X9J,^27^ 6X9K,^27^ 7SFC,^28^ 7SFD,^28^ 7SFE,^28^ 7SFF,^28^ 7SFG,^28^ 7XIB,^29^ 7XI9,^29^ 9V5P,^30^ 9K2W,^31^ and 9K2X^31^). Of these, 4WXX is selected as the template because it is the only human DNMT1 structure to provide structural information for all annotated domains, excluding the first 350 residues. Thus, the structural models of WT DNMT1 and the D490E–P491Y, Y495C, A554V, G589A and V590F variants comprise residues 351–1600 (1250 residues) and correspond to the region resolved in 4WXX, rather than the full-length 1616-residue protein. They include the RFTS, CXXC, ALIK, BAH1, BAH2, and CAT domains (Figure S1). The H++ web server determines the protonation states of protein residues at the physiological pH of 7.4.^32^ The ff19SB force field is used for proteins,^33^ and the SAM parameters are taken from our previous work.^2^ The charges of the Zn coordination polyhedron (Figure S1 and Table S3) are derived from the geometry-optimized structure at the B3LYP level of theory^34^ with the basis set 6-31G­(d).^35^ The restrained electrostatic potential (RESP) fitting method^36^ is employed, together with the Merz–Kollman (MK) scheme.^37^ The van der Waals parameters of the Zn coordination polyhedron are obtained from the previous work by Li et al.^38^ The bonded parameters of the Zn coordination polyhedron are calculated at the same level of theory and obtained using the Seminario method.^39^ All QM calculations are performed using Gaussian09.^40^

The AMBER24 software suite is used to perform MD calculations.^41^ The simulations employ the Particle-Mesh Ewald method^42^ for long-range electrostatic interactions, a 10 Å cutoff for non-bonded interactions, and short-range calculations of PME and Lennard-Jones forces.^43^ The SHAKE algorithm is used to constrain all bonds containing hydrogen atoms.^44^ Periodic boundary conditions are used. Initially, we subject all systems to energy minimization (10,000 steps of steepest descent followed by 10,000 steps of conjugate gradient) to eliminate steric clashes between atoms.

The minimized structures are then solvated in an orthorhombic box using the OPC water model,^45^ ensuring a minimum distance of 12 Å between the solute and the box boundary. To neutralize the system, Na^+^, Cl^–^, and K^+^ ions are added (Li/Merz parameters^46^), with the K^+^ concentration set to mimic intracellular conditions (Table S2). The solvated complexes then undergo a three-stage minimization protocol: (i) restrained minimization (100 kcal/(mol·Å^2^)) across the entire solute, (ii) partially restrained minimization (heavy atoms only), and (iii) unrestrained minimization. Each stage consists of 10,000 steepest-descent steps and 10,000 conjugate-gradient steps. Each system is then heated from 100 K to 310 K over 0.5 ns with a 100 kcal/(mol·Å^2^) restraint applied to the solute heavy atoms. Following this, the system equilibrates at 310 K for 4.5 ns using Langevin dynamics.^47^ The time integration step is 1 fs throughout this phase. Finally, each system undergoes four independent replicates, each 2.5 microseconds long, under NPT conditions (310 K, 1 atm) using a 2-femtosecond time step and randomized initial velocities. Langevin dynamics^47^ and a Monte Carlo barostat^48^ are employed to regulate temperature and pressure, respectively, during NPT calculations. The MD snapshots are saved to the trajectory every 10 ps (i.e., every 100 frames per ns).

### 2.2 Property Analyses

The properties presented here are calculated over an 8.0 μs-long trajectory obtained by combining the 2.0 μs equilibrium trajectory from each of the four independent replicates of the system (Figure S2 and S3):

The *clustering analysis* is performed using the *k*-means algorithm,^49^ based on the RMSD of Cα atoms. We select the optimal number of clusters (*k*) so as to (i) minimize the Davies–Bouldin index (DBI);^50^ (ii) maximize the pseudo-F statistic (pSF);^51^ (iii) identify the inflection point (elbow) in the sum of squares regression (SSR)/total sum of squares (SST) ratio.^52^ If no *k* value fulfills criteria (i), (ii), and (iii), we select a *k* value so as to meet two of the three criteria. If no *k* value satisfies at least two criteria, we select the value that best satisfies one criterion while remaining as close as possible to the optima indicated by the other two, avoiding over-partitioning of the conformational ensembles (Figures S4–S9). Tables S4–S9 summarize the clustering information for each trajectory.

The *radii of gyration* are quantified to evaluate the structural compactness of the system investigated:

1 $$Rg=\langle \sqrt{\frac{1}{N}\sum_{i=1}^{N}{|r_{i}-r_{center}|}^{2}}\rangle$$

Where *N* is the number of atoms, **r**_**i**_ is the coordinate of the atom *i*, and **r**_center_ denotes the coordinate of the geometric center of proteins, <·> indicates averaging over the trajectories.

The *principal component (PC) analysis* ^53^ is performed on the Cα atoms of DNMT1 (1250 residues) using 400,000 frames sampled at 20 ps intervals. The trajectories are first superimposed onto the average reference snapshot to remove overall rotational and translational movements. A covariance matrix is then constructed from the atomic Cartesian coordinate fluctuations and diagonalized to obtain 3750 PCs, corresponding to the 3N degrees of freedom of the 1250 Cα atoms. Each PC is associated with an eigenvalue that represents the variance of the protein motion along that component. The PCs are ranked in descending order according to their eigenvalues, with larger eigenvalues corresponding to motions that explain a greater fraction of the total variance.

The *distance fluctuations* ^18^ between residue *i* (*DF_i_*)) and the rest of the protein, made up of 1250 residues, are defined as:

2 $$DF_{i}=\frac{1}{1250}\left(\sum_{1}^{1250}{\langle (d_{ij}-\langle d_{ij}\rangle )}^{2}\rangle \right)$$

where *d*_*ij*_ denotes the Cα distance of residue *i* and *j* in any single MD snapshot, and ⟨·⟩ means the average over the trajectories. The non-normalized and normalized changes in *DF_i_* (Δ*DF_i_*) between variants and the WT DNMT1 read (eqs 2 and 3, respectively):

3 $$\Delta DF_{i}=DF_{i,\mathrm{variant}}-DF_{i,\mathrm{WT}}$$

4 $$\Delta DF_{i}\left(\%\right)=\frac{DF_{i,\mathrm{variant}}-DF_{i,\mathrm{WT}}}{DF_{i,\mathrm{WT}}}\times 100\%$$

The higher the values in Δ*DF_i_* (%), the lower the motion coordination of residue *i* with the other residues in the protein variants. This disconnection makes residue *i* more unstable in the variants.^54^ The overall Δ*DF_i_* (*OF*) quantifies the effect on the overall stability:^54^

5 $$OF=\frac{1}{N}\sum_{i=1}^{N}\Delta DF_{i}$$

The *allosteric communication* ^19^ is calculated to assess the allosteric importance of each residue within DNMT1. We first model the protein as a residue interaction network. In this network, nodes represent amino acid residues and edges represent physical interactions, which are defined by two criteria: van der Waals contacts and hydrogen bonds. The former are defined as occurring when the minimum interatomic distance between any two carbon atoms is 5 Å or less, while the latter are considered to be formed when the donor (D)–acceptor (A) distance is 3.2 Å or less and the D–H...A angle is 125° or greater.

The network topology permits multiple edges between a residue pair if multiple interaction types exist. We then calculate the Betweenness Centrality (*BC*) and Node Correlation Factor (*NCF*) for each node. *BC* reflects the centrality of a node in signal transfer efficiency.^19^ To minimize noise from transient interactions during MD simulations,^19^ edges with occupancy < 50% are excluded from the *BC* calculation. Conversely, *NCF* quantifies the correlation between the conformational dynamics of residue *i* and its local neighbors.^19^ As *NCF* naturally downweights spurious interactions through regional connectivity and mutual information,^19^ all edges are retained for this calculation. Finally, a combined allosteric score for each residue *i* is derived by averaging the max-min normalized *BC* and *NCF* values:^55^

6 $$AS_{i}=\frac{NBC_{i}+NNCF_{i}}{2}$$

Higher scores indicate a more important role in the protein’s intramolecular communication.^55^ The normalized difference *AS_i_* quantifies the effect on the allosteric importance:

7 $$\Delta AS_{i}\left(\%\right)=\frac{AS_{i,\mathrm{variant}}-AS_{i,\mathrm{WT}}}{AS_{i,\mathrm{WT}}}\times 100\%$$

The CPPTRAJ^56^ is used for all property calculations, except for Δ*DF_i_* (computed using distance_fluctuation.py^18^), and ΔA*S_i_* (calculated using SenseNet^19^ within the Cytoscape platform^57^).

## 3 Results and Discussion

All systems remain globally folded throughout the four independent simulations (Figure 1), which is consistent with the experimental findings.^14,15,17^ The RMSD values fluctuate around an average of 5–7 Å after 0.5 μs (Figure S2). The large RMSD value is primarily due to the movement of the RFTS and CXXC domains (Figure S3): excluding their contributions, the values oscillate around 4.5 Å. The last 2.0 μs of each simulation are combined for further analysis (8.0 μs for each system). The RMSD between the most representative structures, namely the central structures of the most populated clusters obtained by *k*-means clustering analysis across independent simulations (see section Materials and Methods for details; Tables S4–S9), ranges from 5 Å to 7 Å (Table S10).

**Figure 1:**
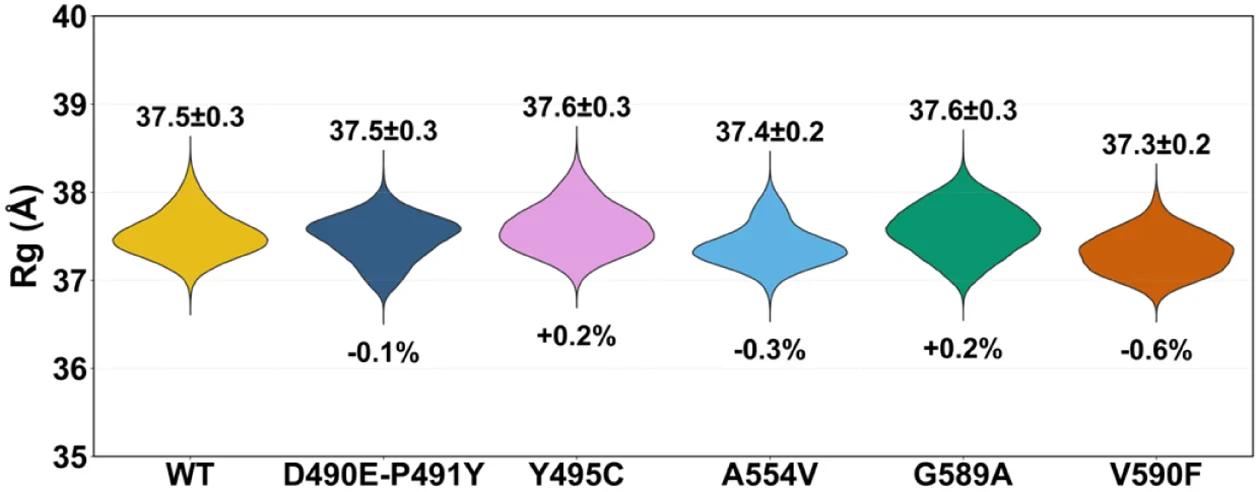
The radius of gyration of the WT DNMT1 and variants. None of the variants significantly affects the structural compactness of DNMT1.

### 3.1 Structural Determinants of ADCA-DN (A554V, G589A, and V590F) Variants and Comparison with *In Vitro* Data

The experimentally observed decreased stability of the DNMT1 variants relative to WT DNMT1 *in vitro,* quantified by reductions in melting temperature,^14,15^ may stem from many factors, including **(i)** mutation-associated disruption of allosteric hubs, which govern long-range intramolecular communications,^58,59^ and **(ii)** mutation-induced changes of structural flexibility.^60^ Other factors include subtle core packing defects, perturbation of local electrostatics,^61^ and internal cavities or hydration of buried regions.^62^ These physicochemical effects are expected to manifest in the protein as altered local dynamics and shifts in the conformational ensemble, and are therefore, to some extent, implicitly reflected in analyses of **(i)–(ii)**.

**(i)** ***Allosteric hubs***: To investigate those, we introduce the “allosteric score” for each residue *i* (A*S_i_*, see section Materials and Methods for details). The larger the A*S_i_*, the more important is the role of residue *i* in long-range allosteric communications.^19,55^ The allosteric hubs are defined as residues with A*S_i_* values greater than 0.25 (40% of the maximum value). This threshold is identified by a threshold-dependent hub-count analysis (Figure S10), which finds an elbow point at 40% of the maximum A*S_i_* value, where the number of hubs begins to stabilize and does not decrease markedly upon further increases in the threshold.

60 Residues out of 1250 (∼5%) are labeled as allosteric hubs (Figure S11). Of these, the CAT domain contains the largest number of hubs (27), followed by the RFTS domain (18). These findings, consistent with the results of a previous 0.2 μs MD simulation of DNMT1 in solution using the AMBER03 force field,^12^ suggest strong allosteric connections between the CAT and RFTS domains. The remaining hubs are distributed among BAH1 (8), BAH2 (3), and CXXC (1) domains, as well as linker regions (3) (Figure S11).

A plot of the normalized difference in A*S_i_* of these hubs between WT DNMT1 and variants (ΔA*S_i_* (%) Figure 2a; see section Materials and Methods for details), shows that as many as 47% to 50% lose their hub character upon mutation (ΔA*S_i_* < −10%), whereas only 12% to 13% feature the opposite (ΔA*S_i_* > 10%). A similar trend is observed for the RFTS domain alone (Figure 2a). Taken together, disrupted allosteric communication, mostly involving the RFTS domain, might contribute to the decreased stability of the mutants.

**Figure 2:**
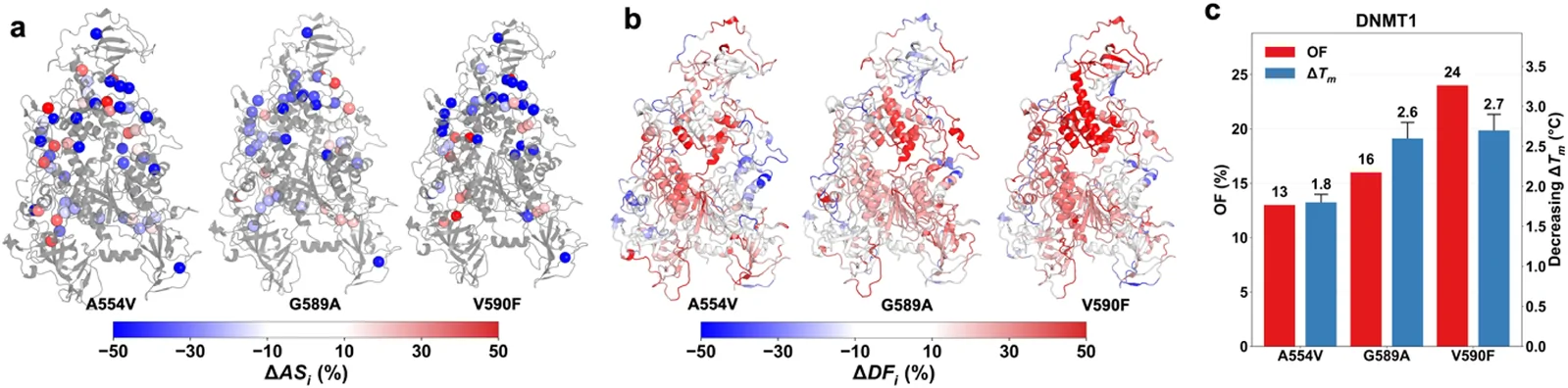
Effect of ADCA-DN-associated variants (A554V, G589A, and V590F) on DNMT1 structural stability. (a) The ΔA*S_i_* value (from −50%, blue to +50%, red) of the residue *i* is related to the allosteric importance on passing from WT DNMT1 to the variants for that residue. The larger the value, the greater the allosteric relevance. The hub residues (A*S_i_ > 0.25;* 40% of the maximum value) are highlighted in spheres (see Figure S10 and Figure S11 for details), while the other regions are coloured in grey. (b) The Δ*DF_i_* score for each residue *i* is related to its flexibility (from −50%, blue to +50%, red). The larger the value, the lower the structural stability upon mutation (from −50%, blue to +50%, red). (c) The *OF* values related to the overall changes of the protein flexibility upon mutations (red), and the melting temperature difference between variants and WT DNMT1 (blue).^14,15^

**(ii) *Structural flexibility***: The distance fluctuation *DF_i_* for each residue *i* of the protein is an index of flexibility and local disorder for that residue.^18,54,55,63^ Then, the normalized difference [Δ*DF_i_* (%)] quantifies the change in structural stability for that residue on passing from WT DNMT1 to the variants^54^ (see section Materials and Methods for details).

A plot of the Δ*DF_i_* (%) values (Figure 2b and 2c) shows that most regions (49%–64%) become more flexible upon mutation, while the opposite occurs for only less than 14%. The remaining regions show negligible changes, that is |Δ*DF*| < 10%. The RFTS domain shows similar values to those of the entire protein (data not shown). Consistently, the overall flexibility changes of the proteins upon mutation (that is, the normalized algebraic sum of the Δ*DF_i_* contributions from all *N* residues, *OF*; see section Materials and Methods for details) are positive for all mutants. Specifically, it increases on passing from A554V (13%) to G589A (16%) and to V590F (24%, Figure 2c). Thus, the calculated overall flexibility of the proteins qualitatively correlates with the reported reductions in melting temperature^14,15^ (Figure 2c). This might suggest that the rigidity of the proteins might play a role in the observed protein stability.

### 3.2 Structural Insights into HSAN1E Variants (D490E-P491Y and Y495C) and Comparison with Data on Cellular Protein Degradation and Cleavage

We attempt to provide a molecular basis also for the experiments on the HSAN1E-associated variants (D490E-P491Y and Y495C DNMT1, see Figures S2 and S3; Tables S1 and S2), by performing MD simulations as we did for the previous systems. These mutants, as mentioned above, cause **(a)** enhanced protein degradation^16^ and **(b)** aberrant protein cleavage.^17^ Of course, comparisons between *in silico* models and cellular outcomes should be interpreted with cautiously, as *in silico* models more closely mimic *in vitro* conditions (as opposed to in cells).

Regarding **(a),** the *OF* values and the percentages of hubs that lose allosteric importance increase from D490E-P491Y (5% for *OF* and 60% for hubs) to Y495C (26% for *OF* and 83% for hubs) (Figure 3a and 3b). These trends correlate positively with reported increases in degradation rates^16^ (Figure 3c). Consequently, both mutations might bias the conformational ensemble toward degradation-prone states by enhancing structural flexibility and/or disrupting allosteric communications.

**Figure 3:**
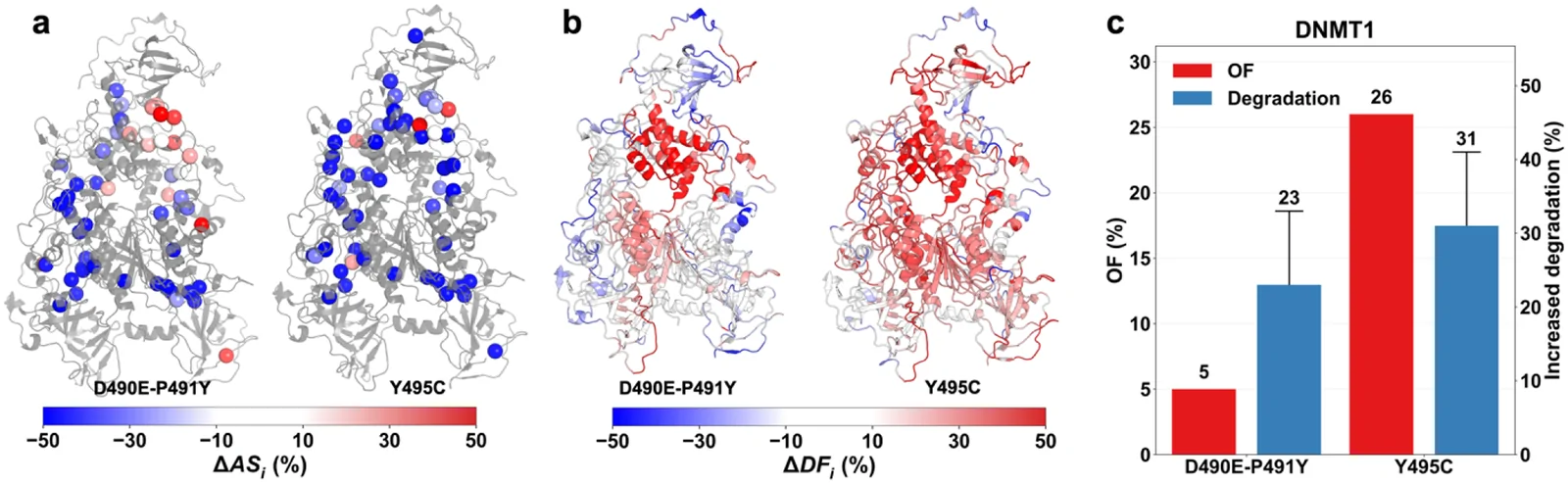
Effect of mutations associated with HSAN1E (D490E-P491Y and Y495C) on the structural stability of DNMT1. (a) Δ*DF_i_* values for the *i*-th residue in both variants. (b) The ΔA*S_i_* of allosteric hubs in both variants. (c) The *OF* (red) and reported increased protein degradation (blue) for these variants.^16^ The color scheme is the same as in Figure 2.

As for (**b),** we note that the cleavage has been proposed to occur near the mutation sites.^17^ In our simulations, the SASA contributions of the mutation sites (where higher values suggest greater solvent exposure) increases from 81(11) to 98(16) Å^2^, on passing from D490 to E490, and from 28(7) to 63(16) Å^2^ for P491 to Y491, as well as from −9 (5) Å^2^ in Y495 to 16 (8) Å^2^ to C495 (Figure 4a–4c). Thus, the mutations might enhance protease accessibility, thereby increasing local susceptibility to aberrant cleavage.^17^ If the increases in solvent exposure observed in our simulations also occur in cells, we can propose a mechanistic hypothesis that connects DNMT1 mutations to the cellular stress responses observed in the two HSAN1E mutations investigated here. Specifically, increased exposure and flexibility at the mutant sites could favor the accumulation of proteolytic fragments, which may increase the burden on proteasomal degradation pathways and contribute to impaired clearance of oxidized proteins under metabolic stress.^64,65^ These processes may be associated with elevated reactive oxygen species levels and mitochondrial dysfunction.^66^ Because we relate residue-level SASA changes (Å^2^) to complex biological outcomes such as proteasomal overload, mitochondrial dysfunction, and oxidative stress cascades, the above statements are at present proposed at the hypothetical level.

**Figure 4:**
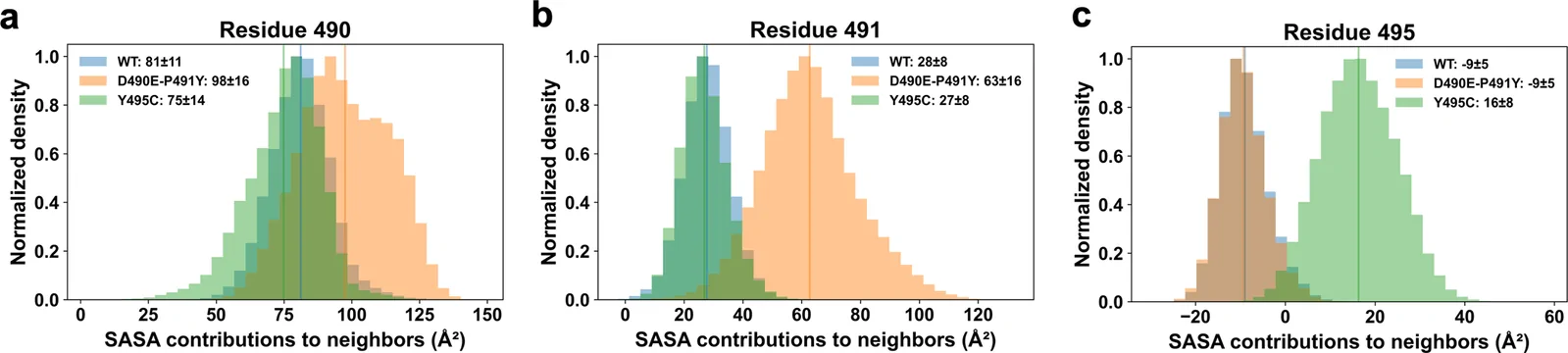
SASA contributions at the mutation sites in WT, D490E-P491Y, and Y495C DNMT1: (a–c) Residues 490, 491, and 495, respectively. Higher contribution values indicate increased solvent exposure at mutant sites. The negative contributions for residue 495 in both the WT and the D490E-P491Y variant are caused by the fact that the residue’s environment is not hydrated (Figure S12). Results for three ADCA-DN mutants are reported in Figure S13, which are identical to those of WT DNMT1.

Patient-derived cells and animal models consistently show mitochondrial dysfunction, elevated oxidative stress, ATP depletion, and widespread DNA hypomethylation, whereas DNMT1 variants do not appear to cause overt structural collapse.^14,15,17^ Our MD simulations suggest that the disease-associated mutations examined here perturb DNMT1 structural dynamics (Figures 2–4), rather than inducing large-scale structural unfolding (Figure 1). At a speculative level, these altered dynamic behaviors, if they also take place *in vivo*, might contribute to impaired DNMT1 function.

### 3.3 Impact of Mutations on DNMT1 Binding to Its Cellular Partners

Mutations altering the structural dynamics of DNMT1 may affect the molecular recognition with its cellular partners. To address this issue, here we use PCA to calculate large-scale motions from MD trajectories.

The two largest motions of WT DNMT1 (PC1 and PC2, accounting for approximately 40% of the overall motion; Figure S14) involve the RFTS domain, the loop linker between BAH2 and CAT, and the flexible loops connecting the CXXC and BAH1 domains (see Movies S1 and S2). The first motion is the opening of the RFTS N-lobe relative to the C-lobe (Movie S1)—a motion similar to that observed in a previous MD study on the 0.2 μs timescale.^12^ PC2 involves loop swinging within the RFTS domain (Movie S2). The variants exhibit markedly different large-scale PC1 and PC2 motions (Figure S14 and Table S11; see Supporting Information for details).

Notably, the large-scale motion of the N-lobe (PC1) observed in WT DNMT1 is markedly reduced in the mutants (Figure S14 and Table S11). At the speculative level, we suggest that the observed disruption to large-scale motion, if it manifests in a cellular environment, could impact the molecular recognition processes of DNMT1 variants interacting with cellular partners (see Table S12). This is the case of UHRF1,^67,68^ H3ub2,^29,69^ and unmethylated (UMDNA)^21^ and hemimethylated (HMDNA) oligonucleotides.^29^ In particular, binding to the first two interactors can promote repositioning of the RFTS domain, thereby exposing the catalytic cavity and facilitating DNA methylation.^29,69^ The disrupted motion in the mutants (Figure S14 and Table S11) might interfere with this conformational coupling, potentially hindering the opening of the DNA cavity in the CAT domain and thereby altering this regulatory mechanism. The same is true for the latter two: the interactors promote RFTS rearrangements, thereby enabling the formation of productive DNA-bound states.^21,70^ It should be noted that post-mitotic neurons rely heavily on DNMT1 for proper DNA methylation patterns.^2,3,71^ Therefore, even subtle reductions in enzymatic or regulatory efficiency may gradually shift the balance toward passive or active demethylation. This interpretation is consistent with the delayed emergence of neurological symptoms despite the presence of DNMT1 mutations from early development,^10,17^ although further validation is needed. Similarly, global hypomethylation in patient induced pluripotent stem cell-derived neurons,^10^ aberrant methylation signatures in patient-derived material,^72^ and loss of imprinted differentially methylated regions in heterozygous mouse models^73^ are consistent with a progressive accumulation of methylation defects.

In addition, the disrupted recognition mechanism of UHRF1 and H3ub2 by DNMT1 may further affect the DNMT1 engagement with higher-order chromatin assemblies. Repressive chromatin domains, including those organized by UHRF1 and H3ub2, are increasingly recognized as multivalent protein networks with condensate-like properties.^74^ In this context, mutations that affect DNMT1 recognition of these partners could, in principle, influence DNMT1 incorporation into or retention within dense regulatory chromatin environments.

Based on these observations, one potential hypothesis is that residual WT DNMT1 activity initially supports the maintenance of baseline methylation but becomes insufficient over time to fully counterbalance ten-eleven translocation-mediated demethylation, stochastic epigenetic drift, or other demethylating processes. Longitudinal methylation profiling in pre-symptomatic mutation carriers, together with direct assays of the effect of the mutations on DNMT1 chromatin recruitment and retention, will be required to test this temporal model.

## 4 Conclusions

Our simulation reveal a clear impact of the ADCA-DN- and HSAN1E-linked mutations on DNMT1 structural dynamics. Our simulations suggest that the three ADCA-DN mutations, A554V, G589A, and V590F, reduce the overall thermal stability of DNMT1 by increasing structural flexibility and disrupting allosteric communication (Figure 2). The two HSAN1E mutations, D490E-P491Y and Y495C, increase structural flexibility and diminish allostery (Figure 3), similar to the effects observed for the ADCA-DN mutations. These disruptions in structural dynamics may contribute to abnormal protein degradation (Figure 3). Additionally, the increased solvent exposure at the mutant sites of both HSAN1E variants (Figure 4), compared to WT DNMT1, could be key to the aberrant protein cleavage. It should be noted, however, that the *in silico* model is different from the real world of cell biology.

At the speculative level, we nonetheless suggest that the disrupted large-scale motions (**Movies S1** and **S2**, Figure S14) and decreased structural stability (Figures 2 and 3) observed in the DNMT1 variants investigated here might influence the recognition of several partners, provided that these occur in cells. Of course, this implication should be tested in future complex-based simulations or biochemical assays.

The limited size of the available experimental dataset (Figure S1 and Table S1) prevents us from drawing statistically robust conclusions. Therefore, the identified mechanisms should be limited to the aforementioned variants. Nevertheless, the consistency of the observed trend across independent datasets and analysis methods suggests a meaningful relationship between mutation-induced disruption of structural dynamics and aberrant DNMT1 function (Figure 2–4).

In conclusion, our findings suggest that restoring DNMT1 structural stability and/or N-lobe motion may represent a viable, experimentally testable therapeutic strategy for the variants investigated here. Ligands or molecular chaperones that stabilize intramolecular allosteric communication or reinforce the architecture of the RFTS domain, which can be assayed via hydrogen–deuterium exchange mass spectrometry, hold the potential to mitigate the effect of mutations. Importantly, the delayed onset of HSAN1E and ADCA-DN provides a potential window for early intervention. Patient-derived iPSC neurons and heterozygous mouse models offer promising platforms for testing whether stabilizing DNMT1 dynamics can delay or prevent neurodegeneration beyond a metabolic threshold.

## Supplementary Material







## Data Availability

The input and script files for the MD simulations have been uploaded to the Zenodo database (https://doi.org/10.5281/zenodo.19877612). The trajectories have been deposited in the MDPosit IRB node (https://mdposit.mddbr.eu/#/browse?search=SongXie).

## Notes

Mapping of DNMT1 mutations associated with ADCA-DN and HSAN1E (**Figure S1**); backbone RMSD profiles of MD simulations (**Figures S2–S3**); evaluation of clustering quality of each trajectory (**Figure S4–S9**); network analysis of WT DNMT1 (**Figure S10 and S11**); local environment of Y495 in WT DNMT1 (**Figure S12)**; SASA analysis for A554V, G589A, and V590F DNMT1 variants (**Figure S13**); mutations’ effect on the large-scale motion of DNMT1 (**Figure S14);** reported experimental effects of DNMT1 variants **Table S1**); information of simulated system (**Table S2**); parameters of Zn­(II) ions **(Table S3)**; clustering information of each system (**Table S4–S9**); variation analysis of independent simulations (**Table S10**); similarity of essential dynamics across different system (**Table S11**); known cellular partners of DNMT1 (**Table S12**) (PDF)
WT DNMT1 large-scale motion along with PC1 (Movie S1) (MP4)
WT DNMT1 large-scale motion along with PC2 (Movie S2) (MP4)

## Abbreviations

DNMT1: DNA methyltransferase 1
ADCA-DN: autosomal dominant cerebellar ataxia–deafness and neuropathy
WT: wild-type
HSAN1E: hereditary sensory and autonomic neuropathy type 1E
SAM: S-adenosyl-L-methionine
NDR: N-terminal disordered region
RFTS: replication foci-targeting sequence
AILK: autoinhibitory linker
BAH: bromo-adjacent homology domains
CAT: C-terminal catalytic domain
MD: molecular dynamics
UHRF1: ubiquitin-like containing PHD and RING finger domains 1 (UHRF1)
H3ub2: ubiquitinated histone H3
SASA: solvent accessible surface area
PCA: principal component analysis
*DF*: distance fluctuations
*AS*: allosteric scores
Rg: radii of gyration
*BC*: betweenness centrality
*NCF*: node correlation factor
*OF*: overall fluctuations
UMDNA: unmethylated oligonucleotides
HMDNA: hemimethylated oligonucleotides
SSR: sum of squares regression
SST: total sum of square.
