# Supplementary material for: Disease-Associated Mutations Impact DNA Methyltransferase 1 Function through Dynamic Allosteric Effects and Solvent Exposure
Source: J Chem Theory Comput. 2026 Aug 21;22(17):9179–87. doi: 10.1021/acs.jctc.6c00982 (PMC13564358; doi:10.1021/acs.jctc.6c00982)
Supplement: Supplementary file 1 [file ct6c00982_si_001.pdf]

Disease-associated Mutations Impact DNA Methyltransferase 1 Function through Dynamic Allosteric Effects and Solvent Exposure

Song Xie[a,b], Can Bora Yildiz[c], Paolo Ruggerone[d], Ke Zuo[e], Geraldine Zimmer-Bensch[c], and Paolo Carloni[a,b,*]

[a]Institute of Neuroscience and Medicine (INM-9) Computational Biomedicine, Forschungszentrum Jülich GmbH, 52428 Jülich, Germany.

[b]RWTH Aachen University, Department of Physics, 52074 Aachen, Germany.

[c]RWTH Aachen University, Department of Neuroepigenetics, Institute of Zoology (Biology 2), Worringerweg 3, 52074 Aachen, Germany.

[d]Department of Physics, University of Cagliari, Cittadella Universitaria, S.P.Monserrato-Sestu km 0.700, I-09044 Monserrato (CA), Italy

[e]College of Pharmacy (International Academy of Targeted Therapeutics and Innovation), Chongqing University of Arts and Sciences, 402160 Chongqing, PR China

* *E-mails: zimmer@bio2.rwth-aachen.de and p.carloni@fz-juelich.de*

Supplemental Figures

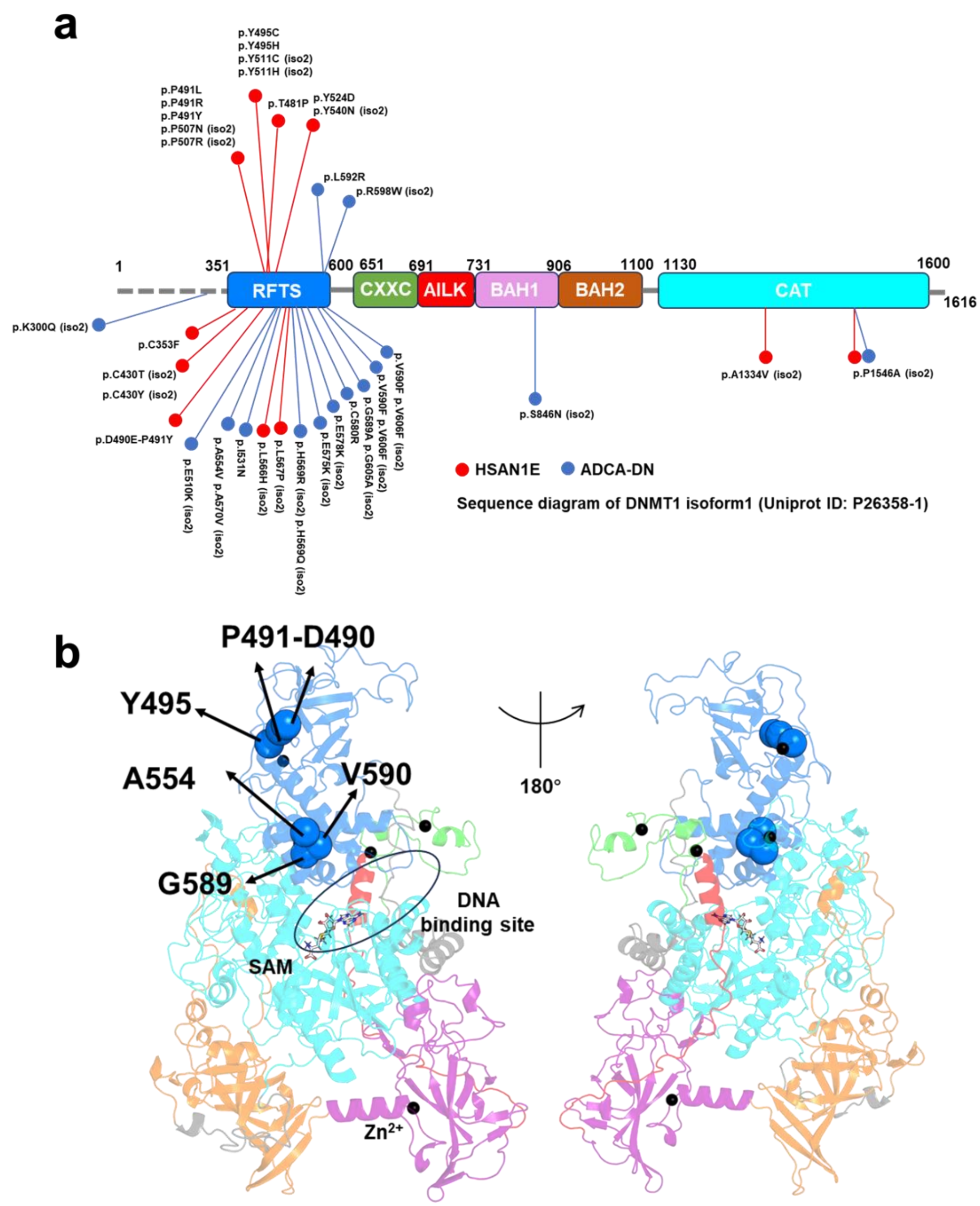

Figure S1. Mapping of DNMT1 mutations associated with autosomal dominant cerebellar ataxia, deafness and narcolepsy (ADCA-DN) and hereditary sensory and autonomic neuropathy type 1E (HSAN1E). (A) Lollipop-style map of mutations associated with ADCA-DN [1-13] (colored in blue) and with HSAN1E [2, 3, 5, 8, 14-22] (colored in red) onto the schematic of the DNMT1 domain architecture. The appropriate isoform, to which each mutation is mapped, is indicated in parentheses. Notably, the patient carrying the p.P1546A mutation exhibits both pathological phenotypes of ADCA-DN and HSAN1E [23]. (B) Cartoon representation of the DNMT1 structural domains: RFTS (blue), CXXC (green), AILK (red), BAH1 (purple), BAH2 (orange), and CAT (cyan). Zn^{2+} ions are rendered as black spheres and S-adenosyl-L-methionine (SAM) as white sticks. The mutations investigated in this study are shown as spheres and labeled by residue number. Numbering follows human DNMT1 isoform 1 (UniProt: P26358-1 [24]); positions originally referring to isoform 2 (P26358-2) have been remapped to isoform 1 for simplification. The model is generated using the SWISS-MODEL web server [25] with the X-ray structure of DNMT1 (PDB: 4WXX [26]) as the template.

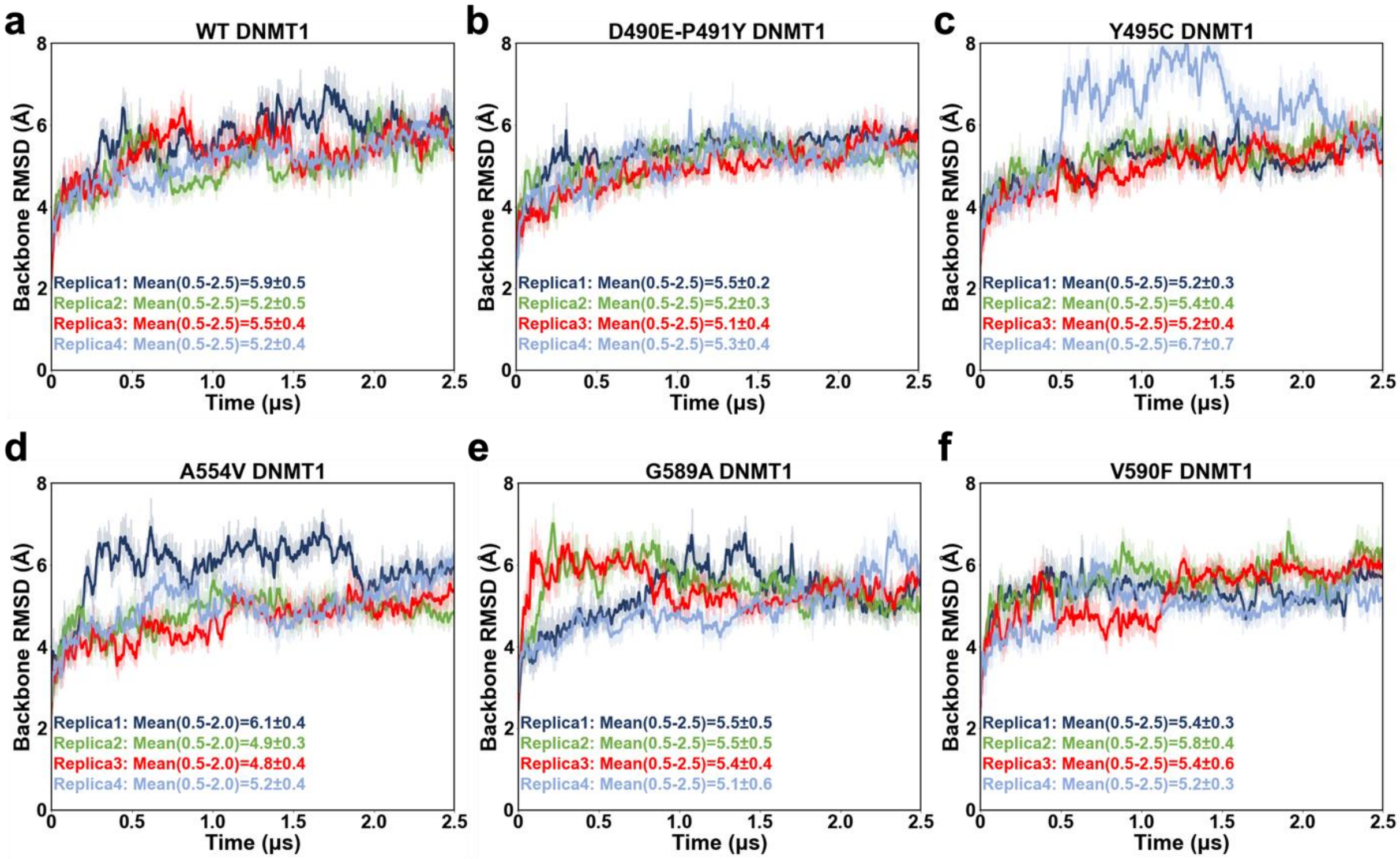

Figure S2. Backbone root-mean-square deviation RMSD profiles of the WT protein and mutants. All trajectories show stability with RMSD fluctuations < 1.0 Å over the final 2.0 µs.

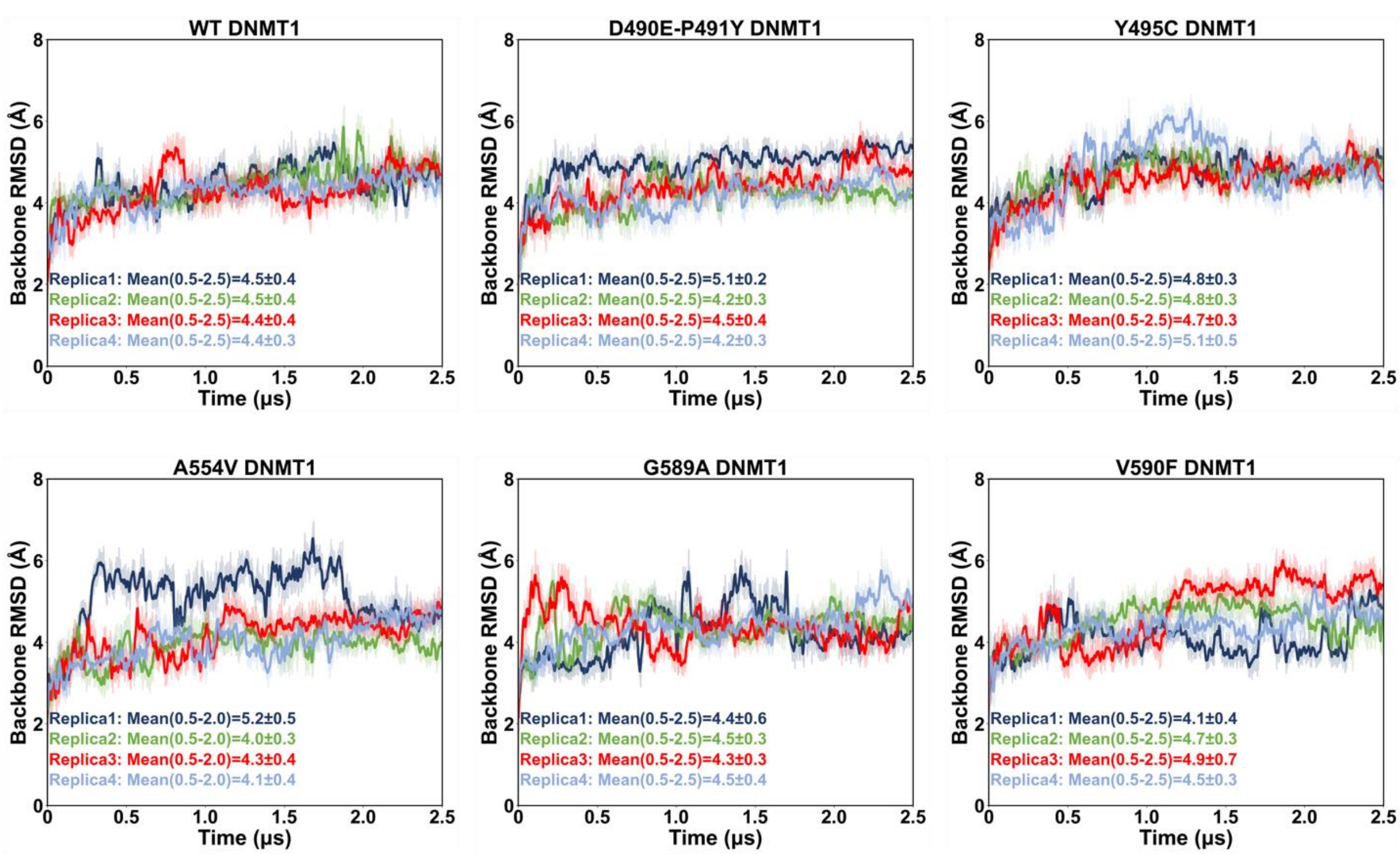

Figure S3. Backbone RMSD profiles of the investigated system, excluding the RFTS and CXXC domains.

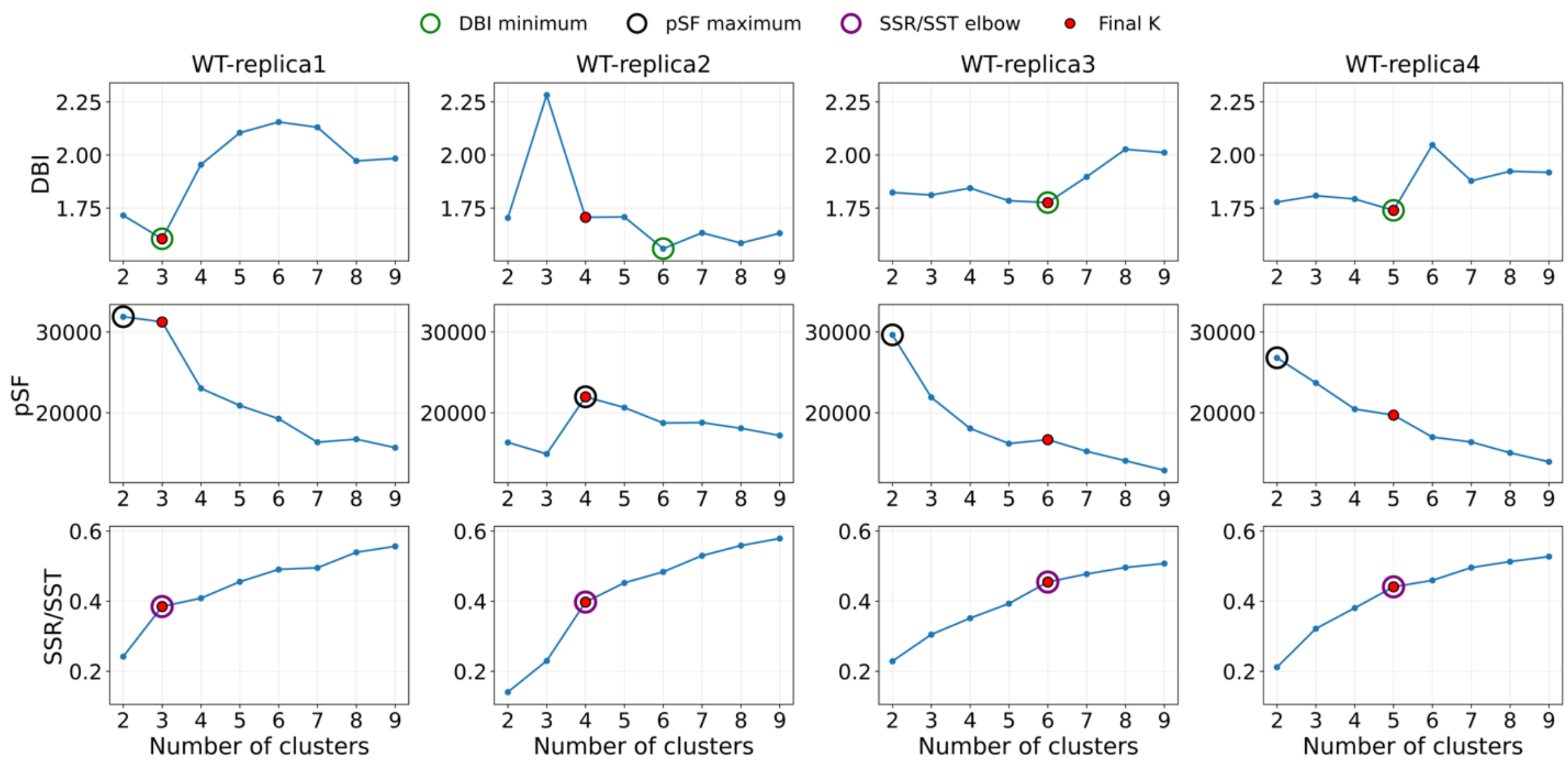

Figure S4. Evaluation of clustering quality for WT DNMT1 trajectories using DBI, pSF, and SSR/SST across different numbers of clusters. The minimum DBI, the maximum pSF, and the elbow of SSR/SST are highlighted by green, black, and purple circles, respectively. The final selected optimal (k) values are indicated by red points (see Materials and Methods for details).

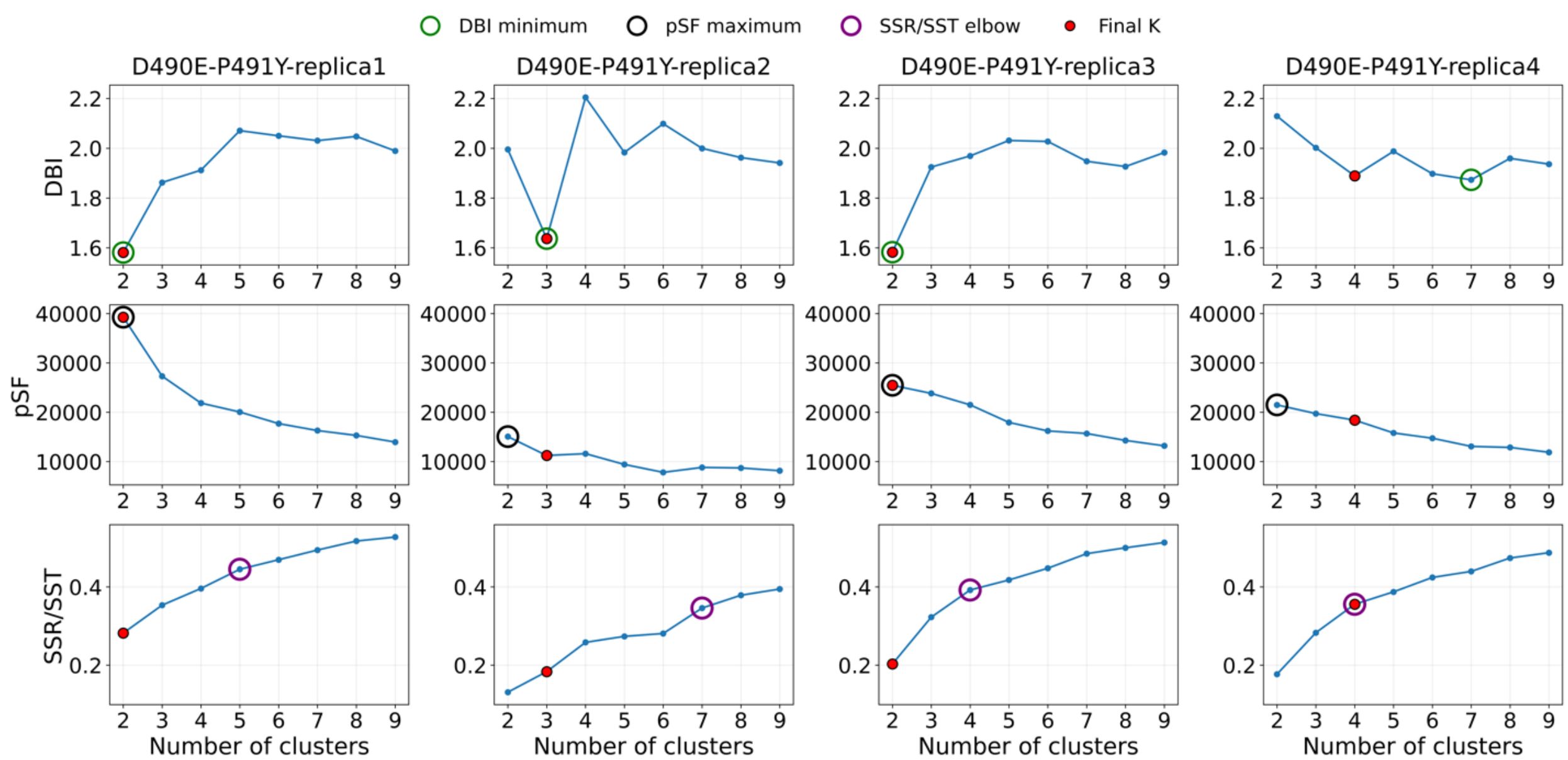

Figure S5. Evaluation of clustering quality for D490E-P491Y trajectories using DBI, pSF, and SSR/SST across different numbers of clusters. The plotting scheme and annotations are the same as those used in **Figure S4**.

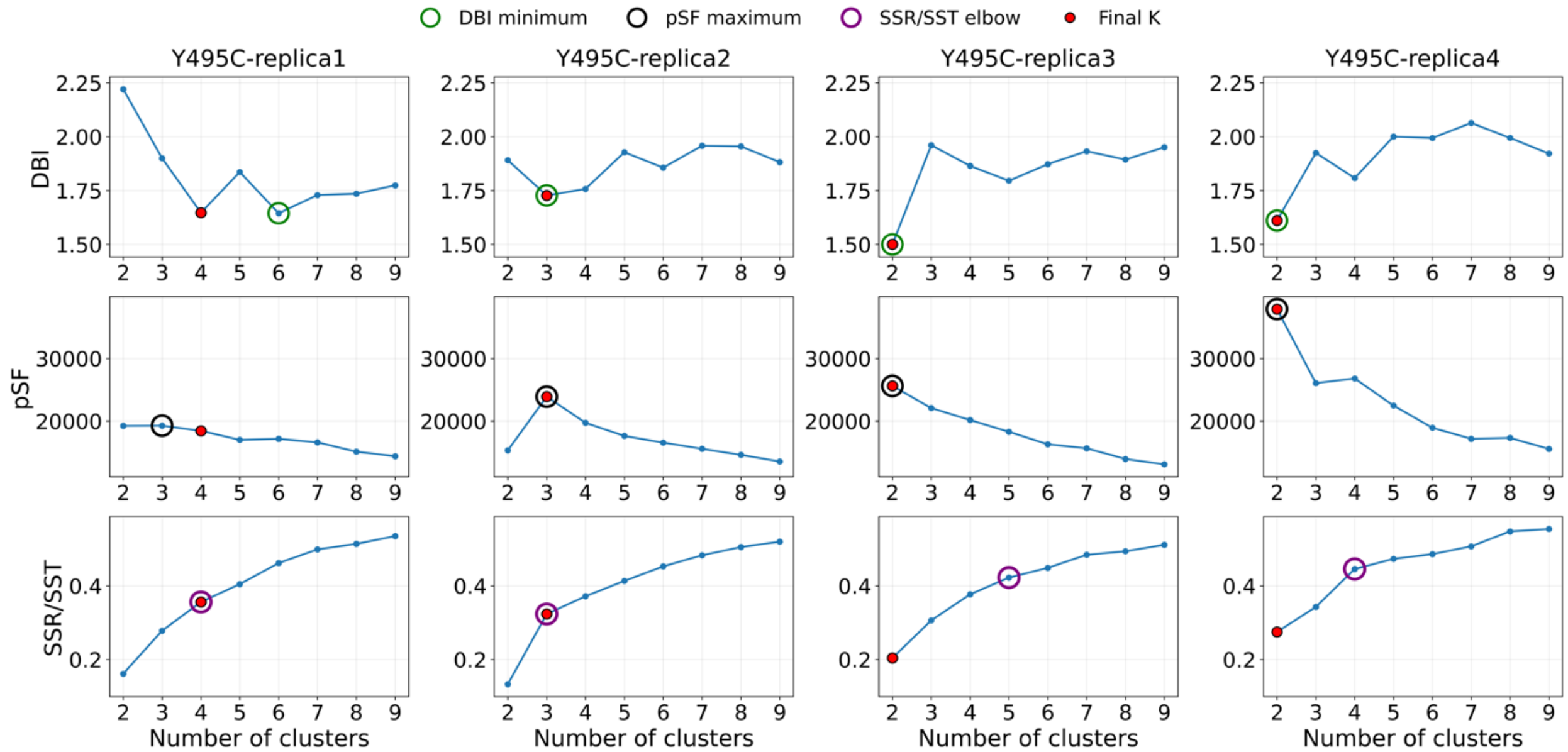

Figure S6. Evaluation of clustering quality for Y495C trajectories using DBI, pSF, and SSR/SST across different numbers of clusters. The plotting scheme and annotations are the same as those used in **Figure S4**.

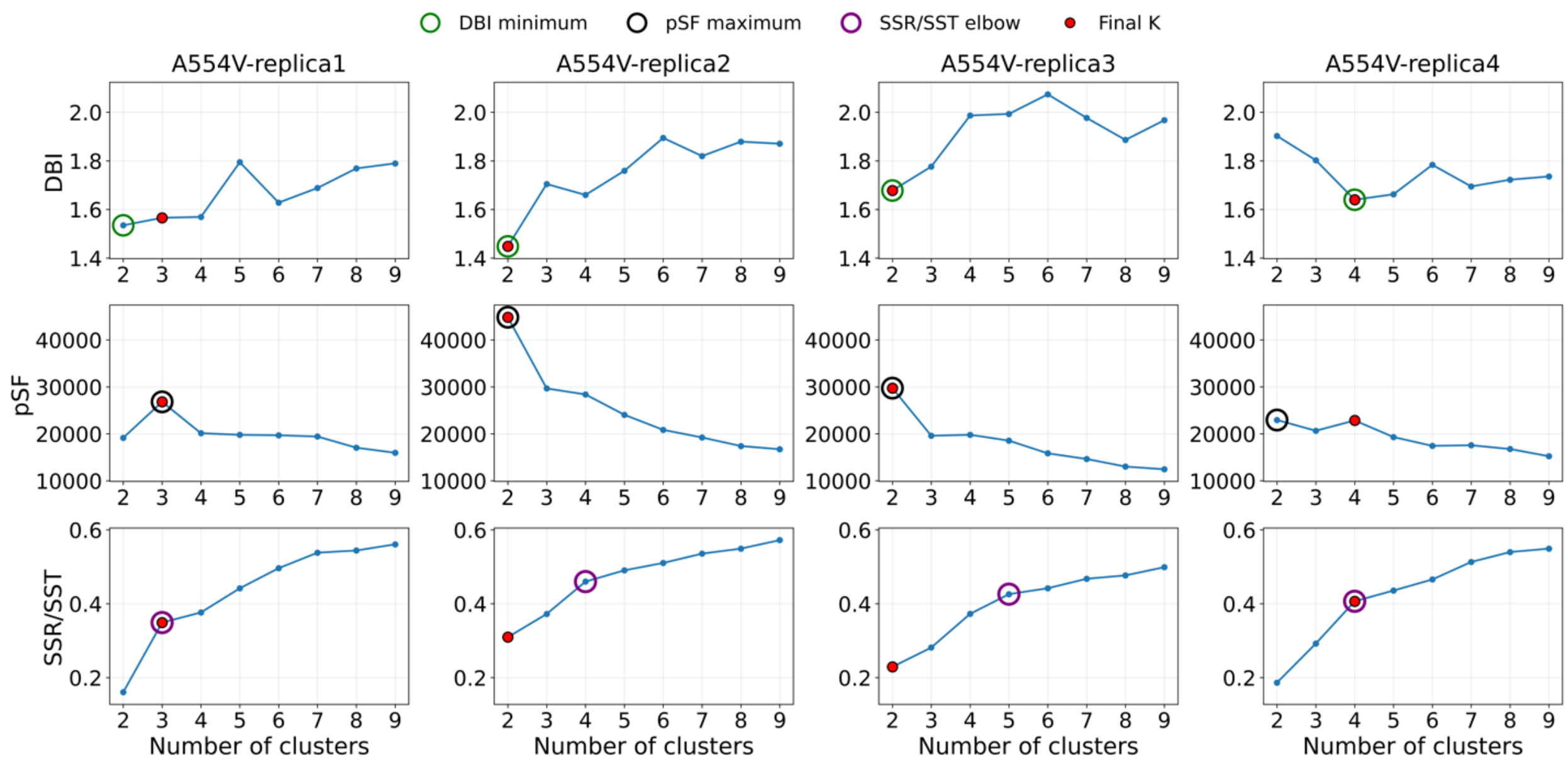

Figure S7. Evaluation of clustering quality for A554V trajectories using DBI, pSF, and SSR/SST across different numbers of clusters. The plotting scheme and annotations are the same as those used in **Figure S4**.

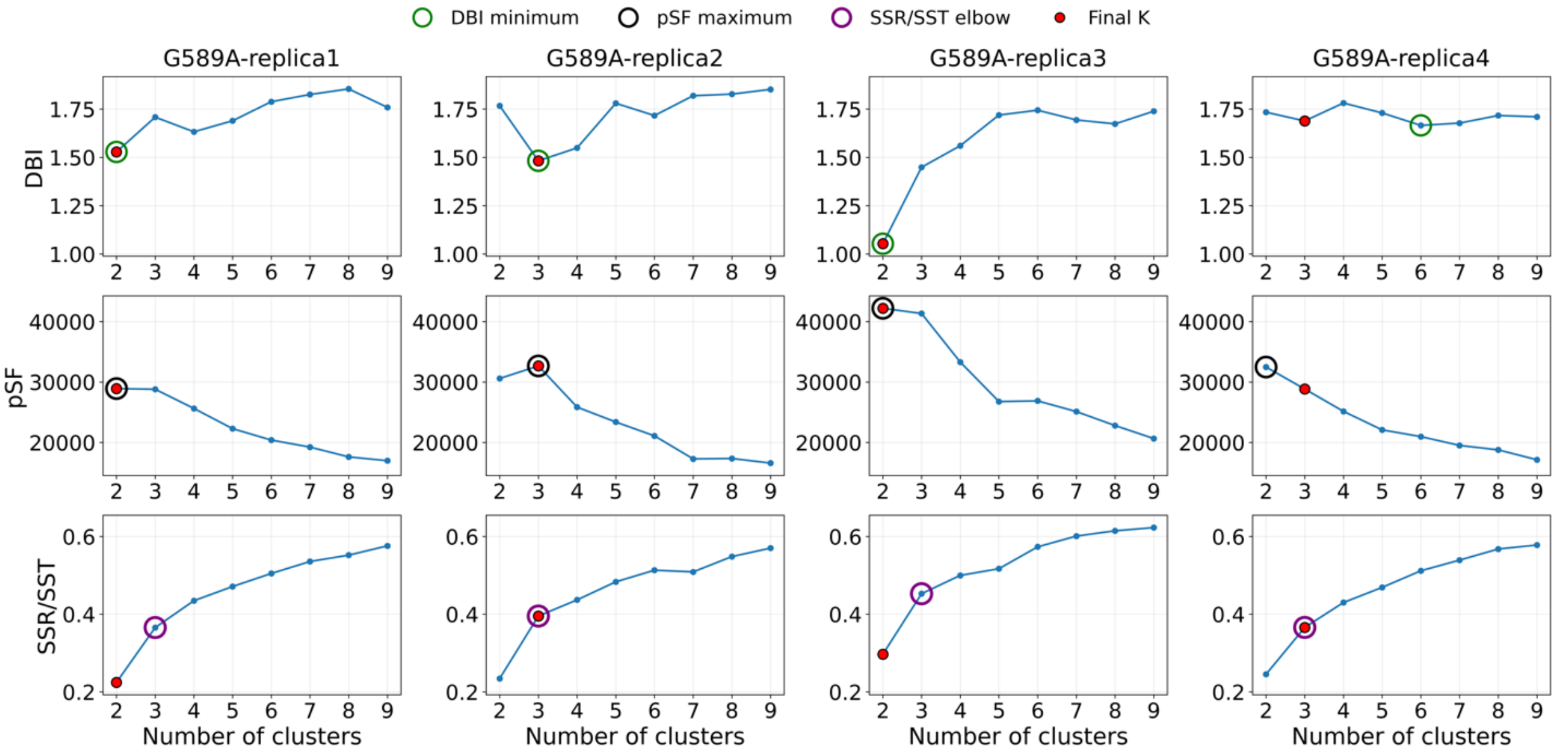

Figure S8. Evaluation of clustering quality for G589A trajectories using DBI, pSF, and SSR/SST across different numbers of clusters. The plotting scheme and annotations are the same as those used in **Figure S4.**

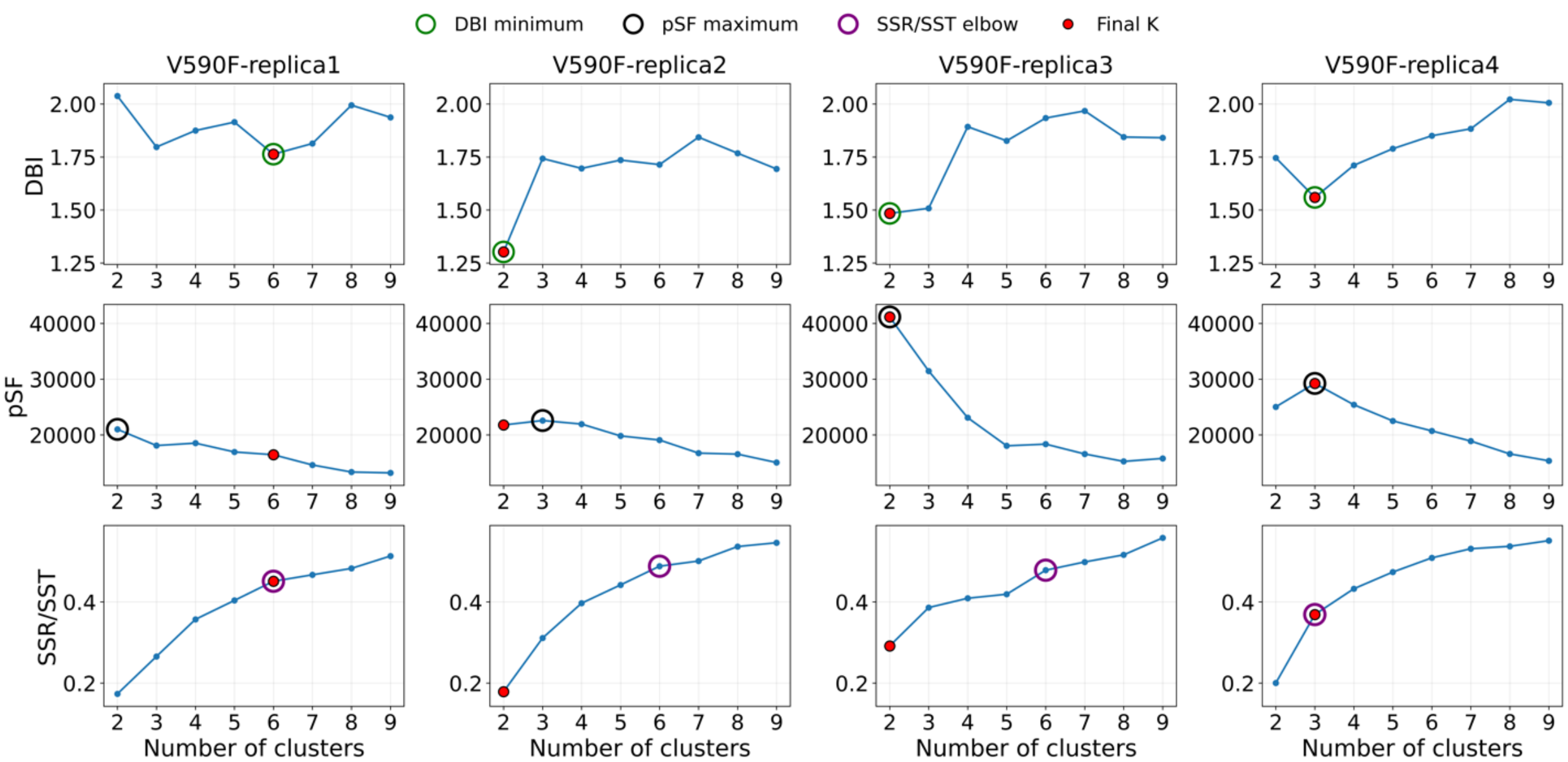

Figure S9. Evaluation of clustering quality for V590F trajectories using DBI, pSF, and SSR/SST across different numbers of clusters. The plotting scheme and annotations are the same as those used in **Figure S4**.

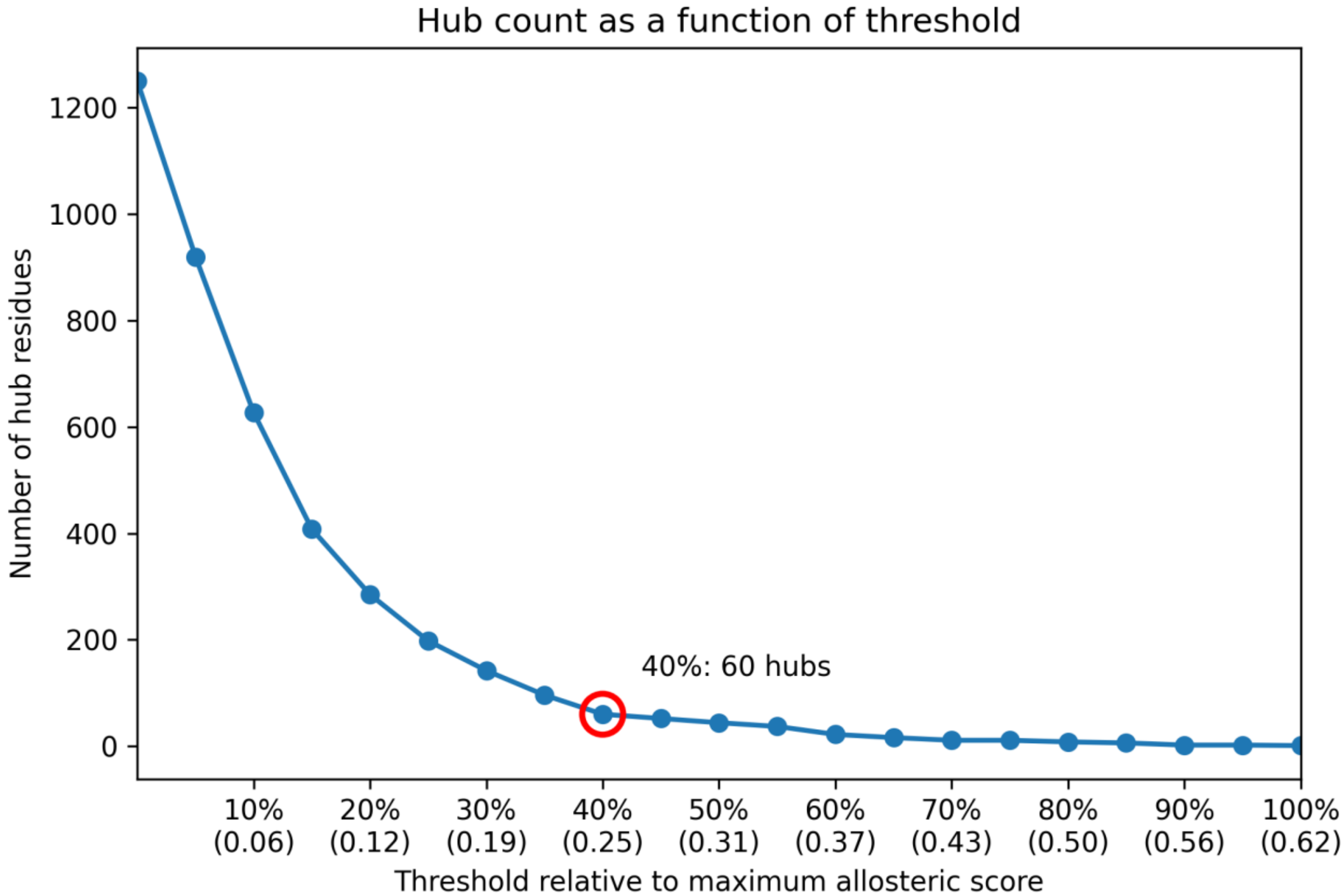

Figure S10. Threshold-dependent hub-count analysis on WT allosteric network. The number of hub residues is plotted against the threshold relative to the maximum (AS_i) value. An elbow-like transition is observed at approximately 0.4 times the maximum value, marked by the red circle, corresponding to 60 hub residues. This threshold is used to define allosteric hubs for further analysis.

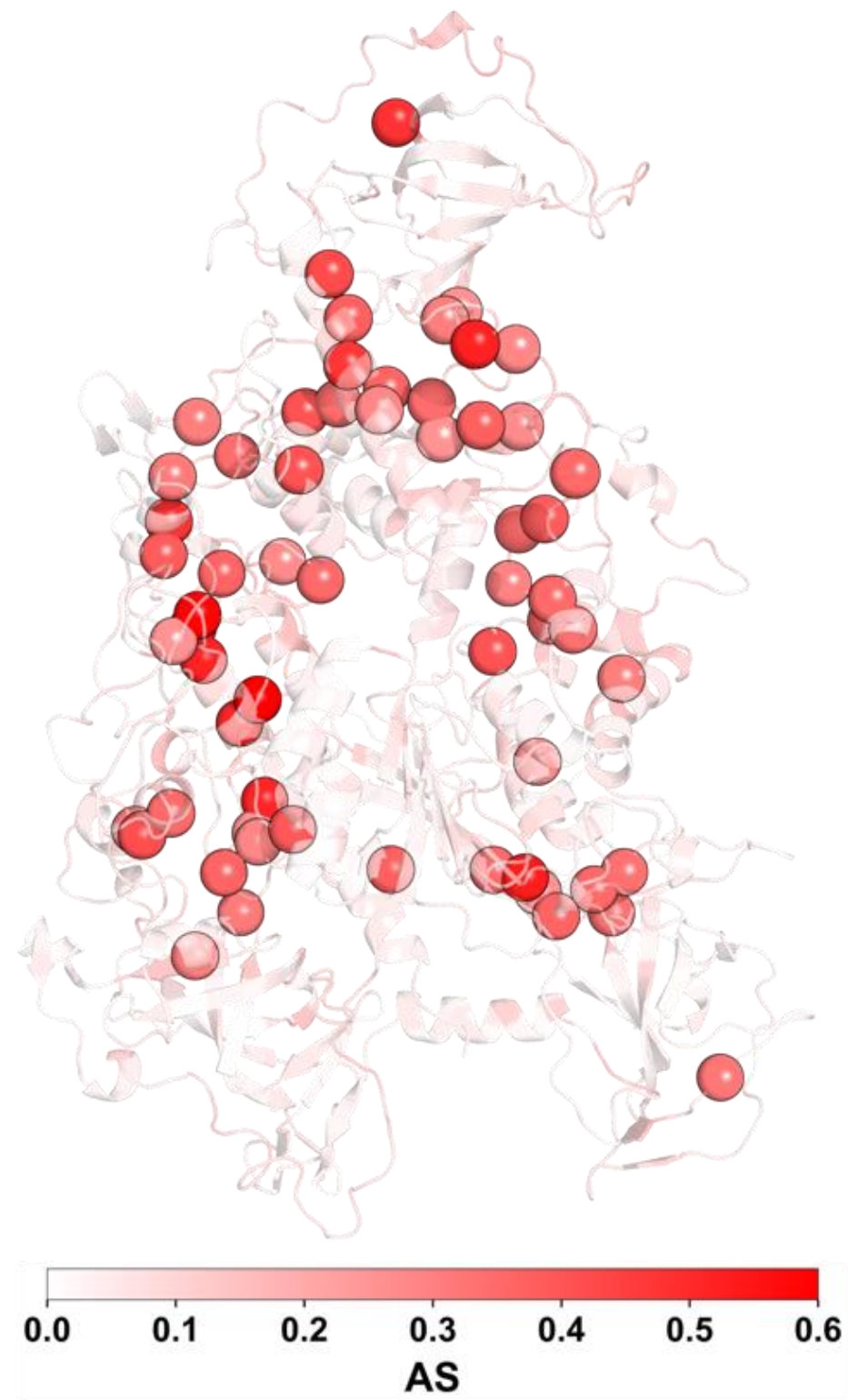

Figure S11. Allosteric scores (AS) for WT DNMT1 residues. Scores range from 0 (white) to 0.6 (red). Residues with the AS scores larger than 40% of the largest value are identified as allosteric hubs and rendered as spheres (see **Figure S10** for details).

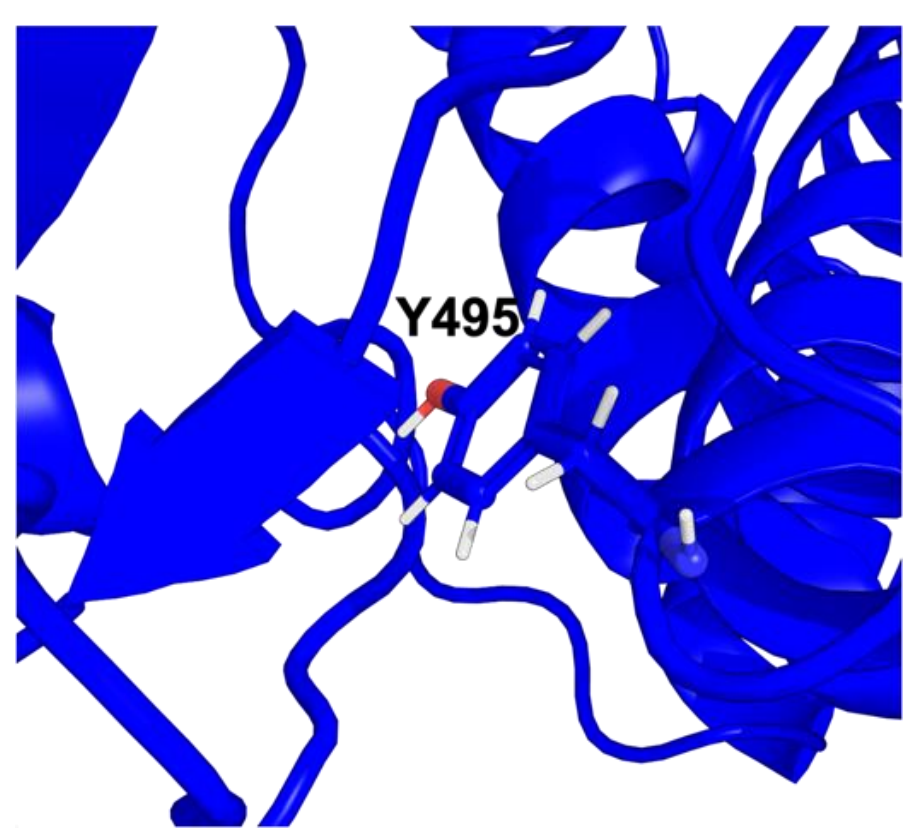

Figure S12. The local environment of Y495 in WT DNMT1. The buried enviroment of Y495 lead to the negative SASA contributions to its neighbouring residues observed in **Figure 4**.

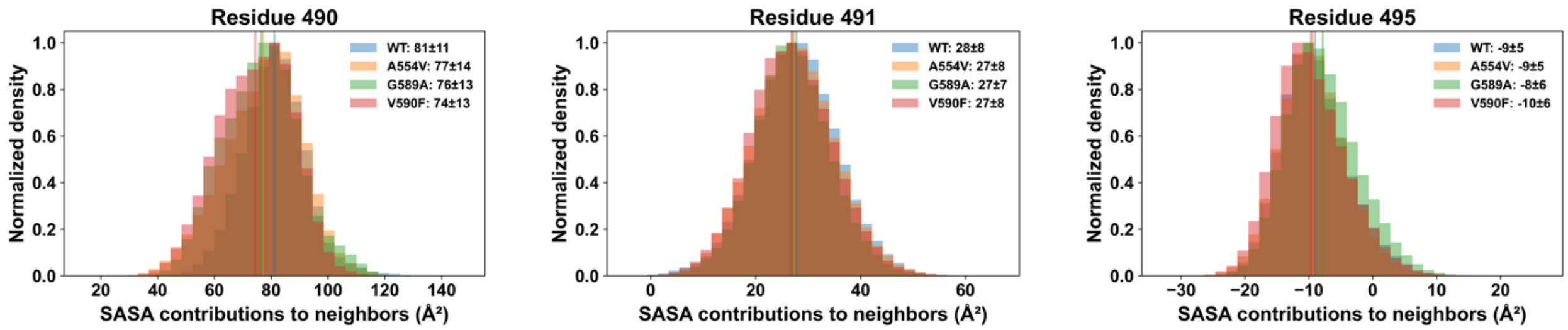

Figure S13. Solvent-accessiable surface aera (SASA) contributions at residues 490, 491, and 495 in WT, A554V, G589A, and V590F DNMT1. These variants do not significantly change SASA contributions of these reidues.

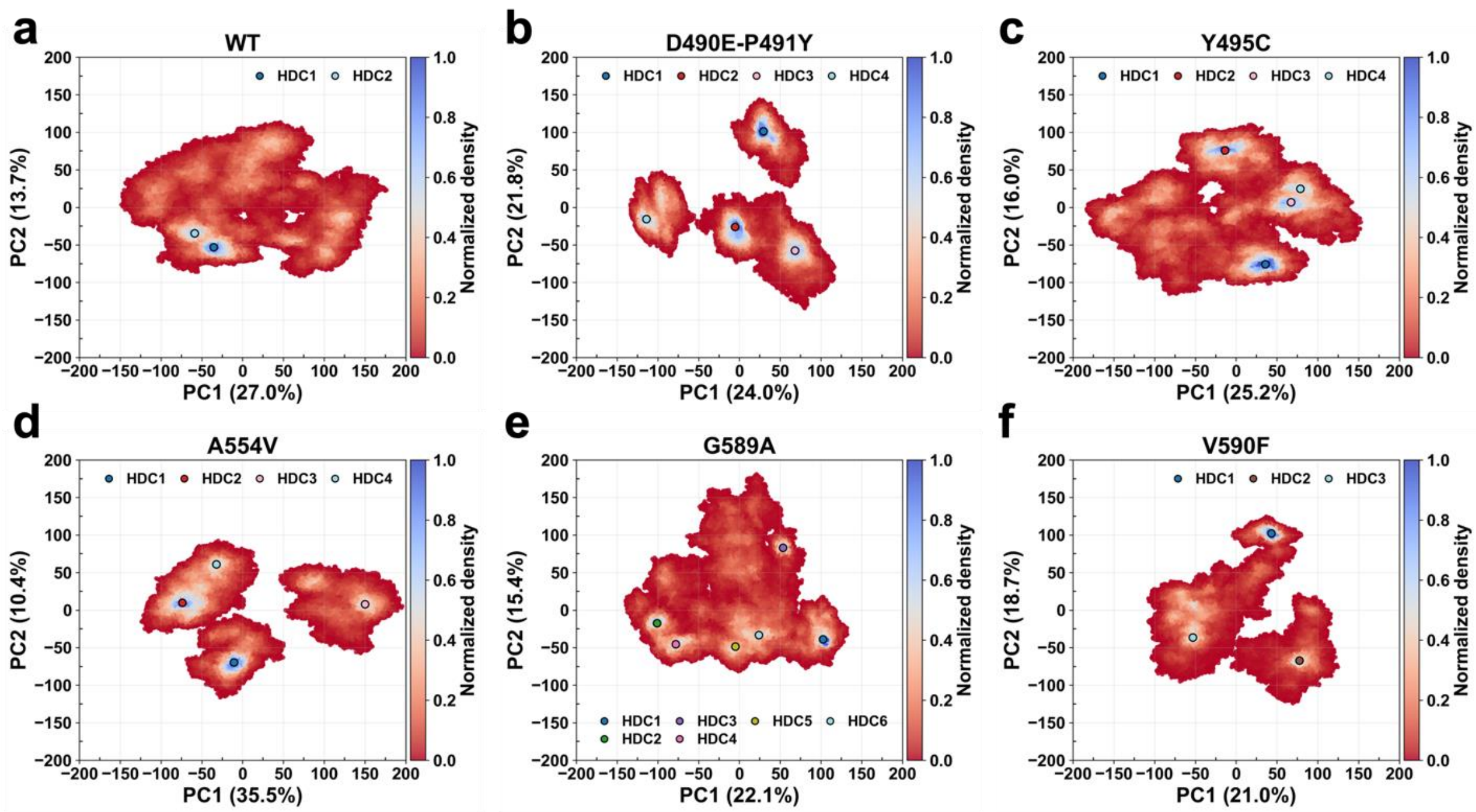

Figure S14. Density surface of the conformational landscape projected onto the first two principal components (PCs) in the motions of WT DNMT1 and variants. Areas are coloured according to normalised conformational density, ranging from 0 (red) to 1 (blue). High-density conformational centres (HDCs), which are defined as regions with a density of 0.5 or greater, are shown as larger dots and labelled in order of density. While the WT protein exhibits a compact landscape characterized by two HDCs, the variants display fragmented landscapes, with a larger number (from 3 to 6) of HDCs.

Supplemental Tables

Table S1. Variants with exerimentally reported effect on DNMT1. The mutations D490E-P491Y, Y495C, A554V, and G589A, and V590F investigated by MD simulations are highlighted in bold face. Note that our simulations provide no insights on protein expression, subcellular localization, and aggregation. Because in the UHRF1- and DNA-DNMT1 complexes DNMT1 is in a different conformation relative to the isolated protein, our MD simulations on DNMT1 in solution cannot provide insights also in binding affinity data, because in formed interactor-DNMT1 complexes the latter may be in a different conformation relative to the isolated protein [27-30], studied here.

Variant	Structural Stability	DNA methylation	Expression	Mis-localized to the Cytosol and Aggregate	Partner Binding
p.C353F [5]	No Data (N.D)	N.D	↓ ≈ 20±10 % (*in vivo*)	G2 phase (*in vivo*)	N.D.
p.T481P [5]	N.D.	N.D	↓ ≈ 60±3 % (*in vivo*)	G2 phase (*in vivo*)	N.D.
**p.D490E-P491Y (double)** [15]	Degradation: ↑~23% ± 10% (Proteasome-mediated) Cleaved by proteases *in vivo*	↓ ≈ 60 ± 2 % (*in vitro*)	N.D.	N.D.	↓ ≈ 70 % interaction with the SAR domain of UHRF1 by RFTS (*in vitro*).
p.P491L [5]	N.D.	N.D	↓ ≈ 40 ± 5% (*in vivo*)	G2 phase (*in vivo*)	N.D.
p.P491Y [18]	N.D.	↓ ≈ 38 ± 4 % (*in vitro*)	N.D.	N.D.	N.D.
**p.Y495C** [5, 15, 18]	Degradation: ↑~31% ± 10% (Proteasome-mediated) Cleaved by proteases *in vivo*	↓ ≈ 55 ± 3 % (*in vitro*)	↓ ≈ 15 ± 5% (*in vivo*)	G2 phase (*in vivo*)	↓ ≈ 66 % interaction with the SAR domain of UHRF1 by RFTS (*in vitro*).
p.Y495H [5]	N.D.	N.D	↓ ≈ 40 ± 5% (*in vivo*)	G2 phase (*in vivo*)	N.D.
p.Y524D [5]	N.D.	N.D	↓ ≈ 40 ± 5% (in cell)	G2 phase (*in vivo*)	N.D.
p.I531N [5]	N.D.	N.D	↓ ≈ 50 ± 3% (*in vivo*)	G2 phase (*in vivo*)	
**p.A554V** [31]	T_m ↓~2 ± 0.1 °C (*in vitro*),	↓ ≈ 60±10 % (*in vitro*)	N.D.	N.D.	↑ ≈ 400 % binding affinity with hemimethylated DNA (*in vitro*).
p.E575K(iso2) [8]	N.D.	↓ ≈ 45 ±10 % (*in vitro*)	N.D.	N.D.	N.D.
**p.G589A** [10]	T_m ↓~3 ± 0.1 °C (*in vitro*)	↓ ≈ 50 ±5 % (*in vitro*)	N.D.	N.D.	↑ ≈ 400 % binding affinity with hemimethylated DNA (*in vitro*).
**p.V590F** [10]	T_m ↓~3 ± 0.1 °C (*in vitro*)	↓ ≈ 50 ±10 % (*in vitro*)	N.D.	N.D.	↑ ≈ 300 % binding affinity with hemimethylated DNA (*in vitro*).

Table S2. Information of simulated system.

System	Charge of complex	Number of K^+ ions	Number of Na^+ ions	Number of Cl^- ions	Number of H_2O molecules
**WT**	-7	186	18	197	49185
**D490E-P491Y**	-7	186	18	197	49392
**Y495C**	-7	186	18	197	49050
**A554V**	-7	186	18	197	49353
**G589A**	-7	186	18	197	49109
**V590F**	-7	186	18	197	49082

Table S3. Zn(II) ion parameters in this study.

	Type	LJ radius (Å)	LJ Depth (kcal/mol)	Charge (e)	GB radius (Å)	GB Screen
**ZN1**	Cys1475-Cys1476-Cys1478-His1502	1.373	0.0118	0.6261	1.500	0.800
**ZN2**	Cys820-Cys892-Cys896-His793	1.373	0.0118	0.8224	1.500	0.800
**ZN3**	Cys664-Cys667-Cys670-Cys686	1.373	0.0118	0.6430	1.500	0.800
**ZN4**	Cys653-Cys656-Cys659-Cys691	1.373	0.0118	0.7860	1.500	0.800
**ZN5**	Cys353-Cys356-Cys414-Cys659	1.373	0.0118	0.6884	1.500	0.800

Table S4. Clustering summary of WT DNMT1 trajectories. The cluster index, in ascending order according to their fraction of the trajectory, is labeled #Cluster. Fraction (%) indicates the percentage of trajectory frames assigned to each cluster. AvgDist and Stdev indicate the average pairwise distance between frames within each cluster and the corresponding standard deviation, respectively. AvgCDist reports the average distance of each cluster to the other clusters. Distance values are reported in Å. Cluster 0 is the most populated cluster.

Trajectory	#Cluster	Fraction (%)	AvgDist	Stdev	AvgCDist
WT-replica1	0	38.7	3.5	0.7	3.5
	1	33.2	3.3	0.6	3.0
	2	28.1	3.3	0.6	3.5
WT-replica2	0	34.9	3.4	0.7	3.1
	1	30.6	3.5	0.8	3.3
	2	22.1	2.8	0.6	3.3
	3	12.4	3.0	0.6	3.7
WT-replica3	0	26.4	3.1	0.5	3.0
	1	22.3	3.2	0.5	3.2
	2	16.5	3.3	0.6	3.1
	3	16.2	3.1	0.5	3.1
	4	10.8	2.8	0.5	3.4
	5	7.9	2.8	0.6	3.3
WT-replica4	0	25.4	2.7	0.4	2.8
	1	24.4	3.0	0.5	2.8
	2	20.4	2.8	0.5	3.1
	3	17.9	2.6	0.4	2.5
	4	12.0	2.8	0.5	2.7

Table S5. Clustering summary of the D490E-P491Y variant trajectories.

Trajectory	#Cluster	Fraction (%)	AvgDist	Stdev	AvgCDist
D490E-P491Y-replica1	0	53.8	3.0	0.5	2.7
	1	46.2	3.1	0.5	2.7
D490E-P491Y-replica2	0	84.3	2.8	0.4	2.6
	1	8.7	2.9	0.7	2.6
	2	7.0	3.1	0.6	2.7
D490E-P491Y-replica3	0	78.0	3.4	0.6	2.9
	1	22.0	3.0	0.6	2.9
D490E-P491Y-replica4	0	30.4	3.1	0.5	2.8
	1	27.0	3.4	0.7	2.4
	2	22.7	2.6	0.4	2.6
	3	19.9	3.1	0.6	3.0

Table S6. Clustering summary of the Y495C DNMT1 trajectories.

Trajectory	#Cluster	Fraction (%)	AvgDist	Stdev	AvgCDist
Y495C-replica1	0	43.2	3.4	0.6	2.8
	1	30.1	2.9	0.5	3.1
	2	17.6	3.2	0.7	3.0
	3	9.1	3.2	0.7	3.2
Y495C-replica2	0	46.0	3.1	0.6	3.0
	1	35.5	3.3	0.6	2.8
	2	18.5	3.6	0.7	3.0
Y495C-replica3	0	80.0	3.7	0.6	3.2
	1	20.0	3.2	0.7	3.2
Y495C-replica4	0	50.9	4.0	0.9	3.5
	1	49.1	3.9	0.7	3.5

Table S7. Clustering summary of the A554V variant trajectories.

Trajectory	#Cluster	Fraction (%)	AvgDist	Stdev	AvgCDist
A554V-replica1	0	56.0	3.6	0.7	3.1
	1	28.8	3.2	0.7	3.8
	2	15.2	3.3	0.7	3.9
A554V-replica2	0	58.4	3.3	0.6	3.3
	1	41.6	3.3	0.7	3.3
A554V-replica3	0	71.2	2.9	0.5	2.6
	1	28.8	3.2	0.6	2.6
A554V-replica4	0	34.7	2.9	0.6	2.9
	1	26.9	3.0	0.6	2.6
	2	22.0	2.8	0.6	2.9
	3	16.4	2.7	0.4	2.9

Table S8. Clustering summary of the G589A variant trajectories.

Trajectory	#Cluster	Fraction (%)	AvgDist	Stdev	AvgCDist
G589A-replica1	0	74.5	3.6	0.9	3.7
	1	25.5	3.6	0.7	4.7
G589A-replica2	0	47.0	3.0	0.7	3.3
	1	35.1	3.2	0.6	3.1
	2	17.9	3.2	0.6	3.6
G589A-replica3	0	82.7	3.2	0.8	4.5
	1	17.3	2.9	0.6	4.5
G589A-replica4	0	42.0	3.3	0.6	3.5
	1	39.5	3.6	0.7	3.0
	2	18.5	3.4	0.9	3.8

Table S9. Clustering summary of the V590F variant trajectories.

Trajectory	#Cluster	Fraction (%)	AvgDist	Stdev	AvgCDist
V590F-replica1	0	25.7	3.2	0.5	2.9
	1	18.4	2.8	0.5	2.6
	2	15.1	3.0	0.6	2.9
	3	14.7	2.9	0.5	2.9
	4	13.1	3.2	0.6	3.3
	5	12.9	2.8	0.6	3.9
V590F-replica2	0	87.8	4.0	0.8	4.0
	1	12.2	3.3	0.7	4.0
V590F-replica3	0	68.7	2.9	0.5	3.2
	1	31.3	3.6	0.8	3.2
V590F-replica4	0	46.0	3.0	0.5	2.8
	1	30.7	2.9	0.6	3.1
	2	23.2	3.3	0.7	3.2

Table S10. The RMSD (Å) matrix among the central structures of the clusters that account for the largest fractions of the trajectories across four independent simulations (see Table S4-S9 and Materials and Methods for details).

	**WT-replica1**	**WT-replica2**	**WT-replica3**	**WT-replica4**
**WT-replica1**	0	5.9	6.0	5.4
**WT-replica2**	5.9	0	5.7	5.3
**WT-replica3**	6.0	5.7	0	5.3
**WT-replica4**	5.4	5.3	5.3	0
	**D491E-P491Y-replica1**	**D491E-P491Y-replica2**	**D491E-P491Y-replica3**	**D491E-P491Y-replica4**
**D491E-P491Y-replica1**	0	5.3	6.4	6.1
**D491E-P491Y-replica2**	5.3	0	5.6	4.9
**D491E-P491Y-replica3**	6.4	5.6	0	5.8
**D491E-P491Y-replica4**	6.1	4.9	5.8	0
	**Y495C-replica1**	**Y495C-replica2**	**Y495C-replica3**	**Y495C-replica4**
**Y495C-replica1**	0	5.7	5.4	6.9
**Y495C-replica2**	5.7	0	5.9	5.7
**Y495C-replica3**	5.4	5.9	0	6.4
**Y495C-replica4**	6.9	5.7	6.4	0
	**A554V-replica1**	**A554V-replica2**	**A554V-replica3**	**A554V-replica4**
**A554V-replica1**	0	6.4	6.2	6.9
**A554V-replica2**	6.4	0	5.0	4.8
**A554V-replica3**	6.2	5.0	0	4.8
**A554V-replica4**	6.9	4.8	4.8	0
	**G589A-replica1**	**G589A-replica2**	**G589A-replica3**	**G589A-replica4**
**G589A-replica1**	0	5.7	5.8	5.6
**G589A-replica2**	5.7	0	6.7	4.9
**G589A-replica3**	5.8	6.6	0	5.1
**G589A-replica4**	5.6	4.9	5.1	0
	**V590F-replica1**	**V590F-replica2**	**V590F-replica3**	**V590F-replica4**
**V590F-replica1**	0	5.9	5.9	5.7
**V590F-replica2**	5.9	0	5.6	5.1
**V590F-replica3**	5.9	5.6	0	6.0
**V590F-replica4**	5.7	5.1	6.0	0

Table S11. Similarity of essential dynamics between WT DNMT1 and mutants as measured by weighted Root Mean Square Inner Product (wRMSIP) calculated over the first two principal components.

Variant	wRMSIP
**D490E-P491Y**	0.32
**Y495C**	0.17
**A554V**	0.37
**G589A**	0.31
**V590F**	0.20

Table S12. Known cellular partners of DNMT1.

Number	Cellular partners	Ref
1	Ubiquitin carboxyl-terminal hydrolase 7 (USP7)	32
2	ubiquitin-like proteins via PHD and RING finger domains (UHRF1)	33
3	Dedicator Of Cytokinesis 7 (DOCK7)	34
4	diubiquitinated histone H3 (H3Ub2)	35
5	Histone-lysine N-methyltransferase (EZH2)	36
6	Histone deacetylase 1 (HDAC1)	37
7	Histone deacetylase 2 (HDAC2)	38
8	DNA methyltransferase 1-associated protein 1 (DMAP1)	38
9	Histone-lysine N-methyltransferase (EHMT2)	39
10	DNA methyltransferase 3B (DNMT3B)	40
11	Zinc finger protein (SNAI1)	41
12	Histone-lysine N-methyltransferase (SUV39H1)	42
13	Unmethylated oligonucleotides (UMDNA)	29
14	Hemimethylated oligonucleotides (HMDNA)	43

Supplemental Movies

Movies S1 and **S2** show WT DNMT1 large-scale movement alone with PC1 and PC2, respectively. The movement of PC1 refers to the opening of the N-lobe of the RFTS in relation to the C lobe. PC2 involves swinging the loop within the RFTS region.

References

(1) Algahtani, H.; Shirah, B. A novel mutation in the <italic>DNMT1</italic> gene in a patient presenting with pure cerebellar ataxia. *J Genet Med* **2017**, *14* (2), 71-74. DOI: 10.5734/JGM.2017.14.2.71.

(2) Bi, H.; Hojo, K.; Watanabe, M.; Yee, C.; Maski, K.; Saba, S.; Graff-Radford, J.; Machulda, M. M.; St Louis, E. K.; Humes, I. S.; et al. Expanded genetic insight and clinical experience of DNMT1-complex disorder. *Neurology Genetics 6* (4), e456. DOI: 10.1212/NXG.0000000000000456.

(3) Norvil, A. B.; Saha, D.; Dar, M. S.; Gowher, H. Effect of Disease-Associated Germline Mutations on Structure Function Relationship of DNA Methyltransferases. *Genes* **2019**, *10* (5), 369.

(4) Fox, R.; Gosal, D.; Ealing, J.; Murphy, H. CLINICAL MANIFESTATIONS OF DNMT1 MUTATIONS. *Journal of Neurology, Neurosurgery & Psychiatry* **2015**, *86* (11), e4. DOI: 10.1136/jnnp-2015-312379.71.

(5) Baets, J.; Duan, X.; Wu, Y.; Smith, G.; Seeley, W. W.; Mademan, I.; McGrath, N. M.; Beadell, N. C.; Khoury, J.; Botuyan, M.-V.; et al. Defects of mutant DNMT1 are linked to a spectrum of neurological disorders. *Brain* **2015**, *138* (4), 845-861. DOI: 10.1093/brain/awv010.

(6) Yuan, J.; Higuchi, Y.; Nagado, T.; Nozuma, S.; Nakamura, T.; Matsuura, E.; Hashiguchi, A.; Sakiyama, Y.; Yoshimura, A.; Takashima, H. Novel mutation in the replication focus targeting sequence domain of DNMT1 causes hereditary sensory and autonomic neuropathy IE. *Journal of the Peripheral Nervous System* **2013**, *18* (1), 89-93. DOI: 10.1111/jns5.12012.

(7) C Yuen, R. K.; Merico, D.; Bookman, M.; L Howe, J.; Thiruvahindrapuram, B.; Patel, R. V.; Whitney, J.; Deflaux, N.; Bingham, J.; Wang, Z.; et al. Whole genome sequencing resource identifies 18 new candidate genes for autism spectrum disorder. *Nature Neuroscience* **2017**, *20* (4), 602-611. DOI: 10.1038/nn.4524.

(8) Maresca, A.; Del Dotto, V.; Capristo, M.; Scimonelli, E.; Tagliavini, F.; Morandi, L.; Tropeano, C. V.; Caporali, L.; Mohamed, S.; Roberti, M.; et al. DNMT1 mutations leading to neurodegeneration paradoxically reflect on mitochondrial metabolism. *Human Molecular Genetics* **2020**, *29* (11), 1864-1881. DOI: 10.1093/hmg/ddaa014 (acccessed 6/28/2026).

(9) Chang, K.-C.; Kuo, Y.-C.; Hsueh, H.-W.; Lee, N.-C.; Yang, C.-C.; Hsieh, S.-T.; Chao, C.-C. Autosomal dominant cerebellar ataxia, deafness, and narcolepsy with amenorrhea, subclinical optic atrophy, and electroencephalographic abnormality: A case report. *eNeurologicalSci* **2020**, *21*, 100271. DOI: 10.1016/j.ensci.2020.100271.

(10) Dolen, E. K.; McGinnis, J. H.; Tavory, R. N.; Weiss, J. A.; Switzer, R. L. Disease-Associated Mutations G589A and V590F Relieve Replication Focus Targeting Sequence-Mediated Autoinhibition of DNA Methyltransferase 1. *Biochemistry* **2019**, *58* (51), 5151-5159. DOI: 10.1021/acs.biochem.9b00749.

(11) Indelicato, E.; Zech, M.; Amprosi, M.; Boesch, S. Untangling neurodevelopmental disorders in the adulthood: a movement disorder is the clue. *Orphanet Journal of Rare Diseases* **2022**, *17* (1), 55. DOI: 10.1186/s13023-022-02218-8.

(12) Catania, A.; Peverelli, L.; Tabano, S.; Ghezzi, D.; Lamperti, C. DNMT1-complex disorder caused by a novel mutation associated with an overlapping phenotype of autosomal-dominant cerebellar ataxia, deafness, and narcolepsy (ADCA-DN) and hereditary sensory neuropathy with dementia and hearing loss (HSN1E). *Neurological Sciences* **2019**, *40* (9), 1963-1966. DOI: 10.1007/s10072-019-03859-7.

(13) Mukherjee, A.; Pandey, S. Cerebellar Ataxia without Deafness or Narcolepsy and the Hot Cross Bun Sign—an Unusual Ensemble in DNMT1 Mutation. *Movement Disorders Clinical Practice* **2023**, *10* (8), 1219-1221. DOI: 10.1002/mdc3.13791.
(14) Menon, P. J.; Bogdanova-Mihaylova, P.; McDermott, G.; Crowley, P.; Killeen, R. P.; Alexander, M. D.; O'Dowd, S.; Murphy, S. M. DNMT1-associated sensory neuropathy and cerebellar ataxia: A novel variant and review of genotype–phenotype correlation. *Journal of the Peripheral Nervous System* **2023**, *28* (3), 508-512. DOI: 10.1111/jns.12560.
(15) Klein, C. J.; Botuyan, M.-V.; Wu, Y.; Ward, C. J.; Nicholson, G. A.; Hammans, S.; Hojo, K.; Yamanishi, H.; Karpf, A. R.; Wallace, D. C.; et al. Mutations in DNMT1 cause hereditary sensory neuropathy with dementia and hearing loss. *Nature Genetics* **2011**, *43* (6), 595-600. DOI: 10.1038/ng.830.
(16) Parissis, D.; Christodoulou, K.; Kleopa, K. A. Novel de novo DNMT1 gene mutation associated with hereditary sensory and autonomic neuropathy 1E (HSAN1E). *Neurological Sciences* **2023**, *44* (6), 2199-2201. DOI: 10.1007/s10072-023-06652-9.
(17) Coelho, P.; Oliveira Santos, M. First Portuguese patient presenting with hereditary sensory and autonomic neuropathy type 1E associated with a novel mutation in DNMT1 gene. *Neurological Sciences* **2020**, *41* (5), 1289-1290. DOI: 10.1007/s10072-019-04179-6.
(18) Smets, M.; Link, S.; Wolf, P.; Schneider, K.; Solis, V.; Ryan, J.; Meilinger, D.; Qin, W.; Leonhardt, H. DNMT1 mutations found in HSANIE patients affect interaction with UHRF1 and neuronal differentiation. *Human Molecular Genetics* **2017**, *26* (8), 1522-1534. DOI: 10.1093/hmg/ddx057.
(19) Kikuchi, J. K.; Nagashima, Y.; Mano, T.; Ishiura, H.; Hayashi, T.; Shimizu, J.; Matsukawa, T.; Ichikawa, Y.; Takahashi, Y.; Karino, S.; et al. Cerebellar Ataxia as a Common Clinical Presentation Associated with DNMT1 p.Y511H and a Review of the Literature. *Journal of Molecular Neuroscience* **2021**, *71* (9), 1796-1801. DOI: 10.1007/s12031-020-01784-5.
(20) Choudhury, S.; Anne, A.; Singh, M.; Chaillet, J. R.; Mohan, K. N. DNMT1 Y495C mutation interferes with maintenance methylation of imprinting control regions. *The International Journal of Biochemistry & Cell Biology* **2024**, *169*, 106535. DOI: 10.1016/j.biocel.2024.106535.
(21) Zheng, W.; Yan, Z.; He, R.; Huang, Y.; Lin, A.; Huang, W.; Su, Y.; Li, S.; Zhang, V. W.; Xie, H. Identification of a novel DNMT1 mutation in a Chinese patient with hereditary sensory and autonomic neuropathy type IE. *BMC Neurology* **2018**, *18* (1), 174. DOI: 10.1186/s12883-018-1177-2.
(22) Nishihara, H.; Yuan, J.; Omoto, M.; Ogasawara, J.-i.; Koga, M.; Kawai, M.; Higuchi, Y.; Hashiguchi, A.; Takashima, H.; Kanda, T. Novel mutation in the methyltransferase domain of DNMT1 in a hereditary sensory and autonomic neuropathy patient with hearing loss, cataract, and dementia. *Neurology and Clinical Neuroscience* **2015**, *3* (2), 74-77. DOI: 10.1111/ncn3.145.
(23) Bi, H.; Hojo, K.; Watanabe, M.; Yee, C.; Maski, K.; Saba, S.; Graff-Radford, J.; Machulda, M. M.; St Louis, E. K.; Humes, I. S.; et al. Expanded genetic insight and clinical experience of DNMT1-complex disorder. *Neurology Genetics* **2020**, *6* (4), e456. DOI: 10.1212/NXG.0000000000000456.
(24) Consortium, T. U. UniProt: the Universal Protein Knowledgebase in 2025. *Nucleic Acids Research* **2024**, *53* (D1), D609-D617. DOI: 10.1093/nar/gkae1010 (acccessed 2/11/2026).
(25) Waterhouse, A.; Bertoni, M.; Bienert, S.; Studer, G.; Tauriello, G.; Gumienny, R.; Heer, F. T.; de Beer, T. A P.; Rempfer, C.; Bordoli, L.; et al. SWISS-MODEL: homology modelling of

protein structures and complexes. *Nucleic Acids Research* **2018**, *46* (W1), 296-303. DOI: 10.1093/nar/gky427.
(26) Zhang, Z.-M.; Liu, S.; Lin, K.; Luo, Y.; Perry, J. J.; Wang, Y.; Song, J. Crystal Structure of Human DNA Methyltransferase 1. *Journal of Molecular Biology* **2015**, *427* (15), 2520-2531. DOI: 10.1016/j.jmb.2015.06.001.
(27) Bashtrykov, P.; Jankevicius, G.; Jurkowska, R. Z.; Ragozin, S.; Jeltsch, A. The UHRF1 Protein Stimulates the Activity and Specificity of the Maintenance DNA Methyltransferase DNMT1 by an Allosteric Mechanism. *Journal of Biological Chemistry* **2014**, *289* (7), 4106-4115. DOI: 10.1074/jbc.M113.528893.
(28) Berkyurek, A. C.; Suetake, I.; Arita, K.; Takeshita, K.; Nakagawa, A.; Shirakawa, M.; Tajima, S. The DNA Methyltransferase Dnmt1 Directly Interacts with the SET and RING Finger-associated (SRA) Domain of the Multifunctional Protein Uhrf1 to Facilitate Accession of the Catalytic Center to Hemi-methylated DNA. *Journal of Biological Chemistry* **2014**, *289* (1), 379-386. DOI: 10.1074/jbc.M113.523209.
(29) Song, J.; Rechkoblit, O.; Bestor, T. H.; Patel, D. J. Structure of DNMT1-DNA Complex Reveals a Role for Autoinhibition in Maintenance DNA Methylation. *Science* **2011**, *331* (6020), 1036-1040. DOI: doi:10.1126/science.1195380.
(30) Kikuchi, A.; Onoda, H.; Yamaguchi, K.; Kori, S.; Matsuzawa, S.; Chiba, Y.; Tanimoto, S.; Yoshimi, S.; Sato, H.; Yamagata, A.; et al. Structural basis for activation of DNMT1. *Nature Communications* **2022**, *13* (1), 7130. DOI: 10.1038/s41467-022-34779-4.
(31) Switzer, R. L.; Hartman, Z. J.; Hewett, G. R.; Carroll, C. F. Disease-Associated Mutation A554V Disrupts Normal Autoinhibition of DNMT1. *DNA* **2023**, *3* (3), 119-133. DOI: 10.3390/dna3030010.
(32) Cheng, J.; Yang, H.; Fang, J.; Ma, L.; Gong, R.; Wang, P.; Li, Z.; Xu, Y. Molecular mechanism for USP7-mediated DNMT1 stabilization by acetylation. *Nature Communications* **2015**, *6* (1), 7023. DOI: 10.1038/ncomms8023.
(33) Bostick, M.; Kim, J. K.; Estève, P.-O.; Clark, A.; Pradhan, S.; Jacobsen, S. E. UHRF1 Plays a Role in Maintaining DNA Methylation in Mammalian Cells. *Science* **2007**, *317* (5845), 1760-1764. DOI: doi:10.1126/science.1147939.
(34) Pitschelatow, G.; Kurisu, J.; Kawaue, T.; Zuo, K.; Xie, S.; Weber-Hamacher, C.; Bayer-Kaufmann, C.; Du, J.; Wolff, P.; Nagayama, S.; et al. A cytosolic function of DNMT1 controls neuronal morphogenesis via microtubule regulation. *bioRxiv* **2025**. DOI: 10.1101/2025.10.19.683279.
(35) Ishiyama, S.; Nishiyama, A.; Saeki, Y.; Moritsugu, K.; Morimoto, D.; Yamaguchi, L.; Arai, N.; Matsumura, R.; Kawakami, T.; Mishima, Y.; et al. Structure of the Dnmt1 Reader Module Complexed with a Unique Two-Mono-Ubiquitin Mark on Histone H3 Reveals the Basis for DNA Methylation Maintenance. *Molecular Cell* **2017**, *68* (2), 350-360.e357. DOI: 10.1016/j.molcel.2017.09.037.
(36) Viré, E.; Brenner, C.; Deplus, R.; Blanchon, L.; Fraga, M.; Didelot, C.; Morey, L.; Van Eynde, A.; Bernard, D.; Vanderwinden, J.-M.; et al. The Polycomb group protein EZH2 directly controls DNA methylation. *Nature* **2006**, *439* (7078), 871-874. DOI: 10.1038/nature04431.
(37) Robertson, K. D.; Ait-Si-Ali, S.; Yokochi, T.; Wade, P. A.; Jones, P. L.; Wolffe, A. P. DNMT1 forms a complex with Rb, E2F1 and HDAC1 and represses transcription from E2F-responsive promoters. *Nature Genetics* **2000**, *25* (3), 338-342. DOI: 10.1038/77124.

(38) Rountree, M. R.; Bachman, K. E.; Baylin, S. B. DNMT1 binds HDAC2 and a new co-repressor, DMAP1, to form a complex at replication foci. *Nature Genetics* **2000**, *25* (3), 269-277. DOI: 10.1038/77023.
(39) Lee, G. E.; Kim, J. H.; Taylor, M.; Muller, M. T. DNA Methyltransferase 1-associated Protein (DMAP1) Is a Co-repressor That Stimulates DNA Methylation Globally and Locally at Sites of Double Strand Break Repair. *Journal of Biological Chemistry* **2010**, *285* (48), 37630-37640. DOI: 10.1074/jbc.M110.148536.
(40) Kim, G. D.; Ni, J.; Kelesoglu, N.; Roberts, R. J.; Pradhan, S. Co-operation and communication between the human maintenance and de novo DNA (cytosine-5) methyltransferases. *The EMBO Journal* **2002**, *21* (15), 4183-4195. DOI: 10.1093/emboj/cdf401.
(41) Espada, J.; Peinado, H.; Lopez-Serra, L.; Setién, F.; Lopez-Serra, P.; Portela, A.; Renart, J.; Carrasco, E.; Calvo, M.; Juarranz, A.; et al. Regulation of SNAIL1 and E-cadherin function by DNMT1 in a DNA methylation-independent context. *Nucleic Acids Research* **2011**, *39* (21), 9194-9205. DOI: 10.1093/nar/gkr658.
(42) Fuks, F.; Hurd, P. J.; Deplus, R.; Kouzarides, T. The DNA methyltransferases associate with HP1 and the SUV39H1 histone methyltransferase. *Nucleic Acids Research* **2003**, *31* (9), 2305-2312. DOI: 10.1093/nar/gkg332.
(43) Song, J.; Teplova, M.; Ishibe-Murakami, S.; Patel, D. J. Structure-Based Mechanistic Insights into DNMT1-Mediated Maintenance DNA Methylation. *Science* **2012**, *335* (6069), 709-712. DOI: doi:10.1126/science.1214453.
